# Mechanisms and treatments of neuropathic itch in a mouse model of lymphoma

**DOI:** 10.1172/JCI160807

**Published:** 2023-02-15

**Authors:** Ouyang Chen, Qianru He, Qingjian Han, Kenta Furutani, Yun Gu, Madelynne Olexa, Ru-Rong Ji

**Affiliations:** 1 Center for Translational Pain Medicine, Department of Anesthesiology,; 2 Department of Cell Biology, and; 3 Department of Neurobiology, Duke University Medical Center, Durham, North Carolina, USA.

**Keywords:** Neuroscience, Therapeutics, Lymphomas, Skin cancer

## Abstract

Our understanding of neuropathic itch is limited due to a lack of relevant animal models. Patients with cutaneous T cell lymphoma (CTCL) experience severe itching. Here, we characterize a mouse model of chronic itch with remarkable lymphoma growth, immune cell accumulation, and persistent pruritus. Intradermal CTCL inoculation produced time-dependent changes in nerve innervations in lymphoma-bearing skin. In the early phase (20 days), CTCL caused hyperinnervations in the epidermis. However, chronic itch was associated with loss of epidermal nerve fibers in the late phases (40 and 60 days). CTCL was also characterized by marked nerve innervations in mouse lymphoma. Blockade of C-fibers reduced pruritus at early and late phases, whereas blockade of A-fibers only suppressed late-phase itch. Intrathecal (i.t.) gabapentin injection reduced late-phase, but not early-phase, pruritus. IL-31 was upregulated in mouse lymphoma, whereas its receptor *Il31ra* was persistently upregulated in *Trpv1*-expressing sensory neurons in mice with CTCL. Intratumoral anti–IL-31 treatment effectively suppressed CTCL-induced scratching and alloknesis (mechanical itch). Finally, i.t. administration of a TLR4 antagonist attenuated pruritus in early and late phases and in both sexes. Collectively, we have established a mouse model of neuropathic and cancer itch with relevance to human disease. Our findings also suggest distinct mechanisms underlying acute, chronic, and neuropathic itch.

## Graphical abstract

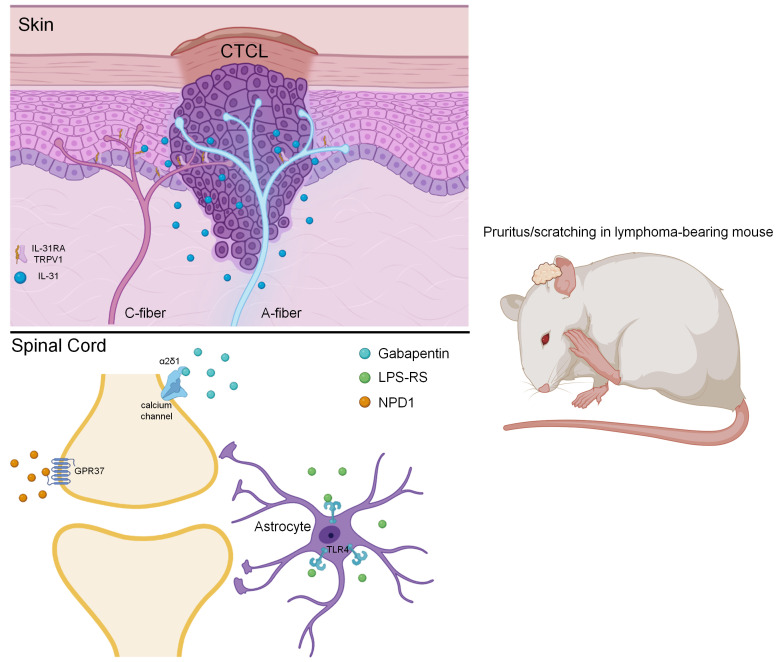

## Introduction

Recent progress in preclinical research has advanced our understanding of molecular and cellular mechanisms of itch (1–3). These advances include the identification of molecular sensors and receptors of itch (4–7), the elucidation of the neurotransmitters and neuromodulators of itch (8–10), and the characterization of neurocircuits of itch (9, 11–17), including those for mechanical itch (13, 18) and opioid-induced itch (19, 20). In addition, glial regulation of itch has also been proposed (21, 22). While acute itch (pruritus) serves as a warning for potential tissue damage, chronic itch leads to sleep disruption and severe skin lesions and is commonly associated with skin diseases as well as systemic disorders, such as liver and kidney diseases, HIV/AIDS, and metabolic disorders (23–25). Antihistamines are the first choice for anti-itch treatment, but most chronic itch conditions are resistant to antihistamines, as histamine-independent mechanisms may dominate in chronic itch. The so-called “itch-scratch-itch” cycle is believed to exacerbate itch. Development of animal models to mimic clinical itch conditions is critical to investigate the molecular mechanisms of chronic itch and test new anti-itch treatments.

A number of animal models have been developed for dermatitis-induced chronic itch, including a dry skin model induced by a mixture of acetone/ether (1:1) and water (AEW), a toxic contact dermatitis model induced by diphenylcyclopropenone (DCP), and an allergic contact dermatitis model induced by 2,4-dinitro-1-fluorobenzene (DNFB), a psoriasis model induced by imiquimod (26), as well as the contact allergen of poison ivy induced by urushiol (27–31). A mouse model of cholestasis was generated via α-naphthylisothiocyanate (ANIT) administration to induce cholestatic pruritus (32, 33). Additionally, spontaneous itch and potentiation of evoked itch were also reported in multiple mouse genetic models (14, 34, 35).

Much like neuropathic pain, neuropathic itch is a result of neuropathy due to damage to the nervous system (36–38), including damage to peripheral nerves, the spinal cord, and the brain. Neuropathic itch could be more common than generally appreciated because most neuropathic pain conditions may have a neuropathic itch component (39). Although several groups have developed genetic models of neuropathic itch (39) and potential treatments for neuropathic itch (40), a clinically relevant model of neuropathic itch caused by peripheral neuropathy, the most common clinical condition of neuropathic itch, is still lacking.

Cutaneous T cell lymphomas (CTCLs) are commonly seen in patients with primary skin lymphomas (41, 42). These patients experience severe and chronic itching (43). We developed a mouse model of CTCL by intradermal inoculation of human Myla cells (from a patient with mycosis fungoides [MF]) in immune-deficient mice (44). In this model, mice not only develop lymphoma but also exhibit remarkable chronic itch that can manifest within several days and last for more than 60 days (44). We found that CTCL resulted in a sustained accumulation of immune cells including mast cells, neutrophils, macrophages, and DCs in the lymphoma. We also demonstrated time-dependent changes of nerve innervations in the epidermis of the lymphoma-bearing back skin: hyperinnervation in the early phase (CTCL day 20) and substantial loss of nerve fibers in the late phase (CTCL days 40 and 60). Accordingly, chronic itch was responsive to gabapentin treatment only in the late phase. We also found obvious, marked nerve innervations in mouse lymphoma. Furthermore, IL-31 signaling pathway was persistently upregulated in the CTCL model and played a critical role in pruritus.

## Results

### Mouse CTCL model is characterized by chronic itch, tumor growth, and accumulation of immune cells.

**Figure 1:**
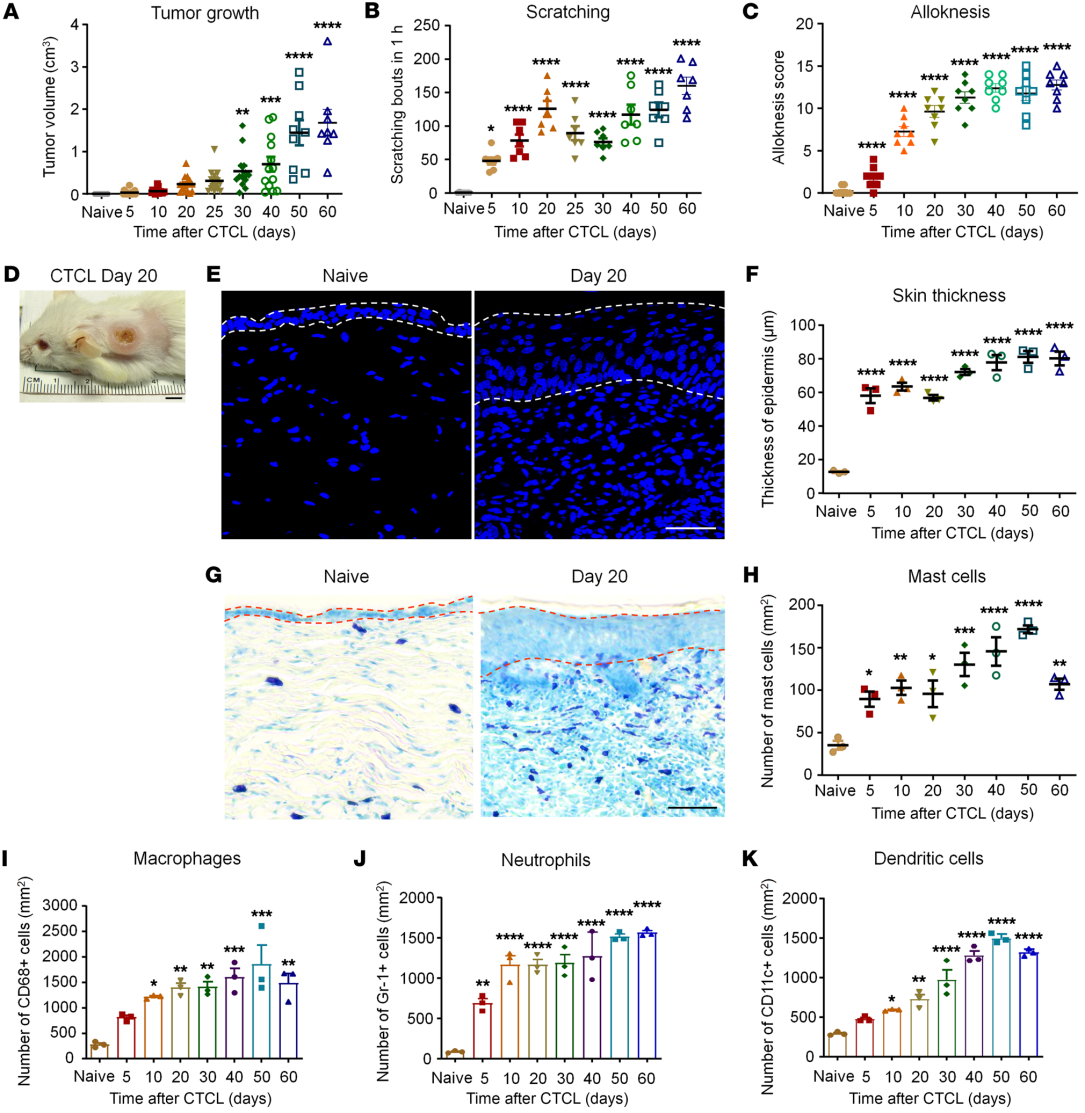
CTCL is associated with tumor growth, immune cell accumulation, and chronic itch. (**A**) Time course of tumor growth in CTCL mice. *n* = 8–18. One-way ANOVA, *F*_(8,__115)_ = 22.36, *P* < 0.0001. (**B**) Time course of scratching bouts in CTCL mice. *n* = 7. One-way ANOVA, *F*_(8,__54)_ = 22.61, *P* < 0.0001. (**C**) Time course of alloknesis in CTCL mice. *n* = 8. One-way ANOVA, *F*_(7,__56)_ = 68, *P* < 0.0001. Alloknesis was induced by an innocuous von Frey filament (bending force = 0.07 g). (**D**) Representative image of lymphoma on day 20. Scale bar: 0.5 cm. (**E**) Representative images of DAPI immunostaining of the back skin of naive and CTCL mice at day 20. Dotted lines show the epidermis. Scale bar: 40 μm. (**F**) Time course of the thickness of the epidermis of CTCL mice. *n* = 3. One-way ANOVA, *F*_(7,__16)_ = 50.07, *P* < 0.0001. (**G**) Representative images of toluidine blue staining for mast cells in the back skin of naive and CTCL mice at day 20. Dotted lines show the epidermis. Scale bar: 50 μm. (**H**) Time course showing the number of mast cells in lymphoma tumors. *n* = 3. One-way ANOVA, *F*_(7,__16)_ = 14.07, *P* < 0.0001. (**I**–**K**) Time course showing the number of CD68^+^ macrophages (**H**), Gr-1^+^ neutrophils (**I**), and CD11C^+^ DCs (**J**). *n* = 3. One-way ANOVA, (**H**) *F*_(7,__16)_ = 8.56, *P* = 0.0002; (**I**) *F*_(7,__16)_ = 29.54, *P* < 0.0001; and (**J**) *F*_(7,__16)_ = 60.65, *P* < 0.0001. Data are expressed as the mean ± SEM. One-way ANOVA with Bonferroni’s post hoc test, *P < 0.05, **P < 0.01, ***P < 0.001, and ****P < 0.0001 versus the naive group.

The murine xenograft model of CTCL was produced by inoculation of human Myla cells (CD4^+^ memory T cells) from a patient with MF (42). Intradermal inoculation of Myla cells (1 × 10^5^ cells/μL, 100 μL) on the nape of the neck of severe combined immunodeficient (SCID) mice (NOD.CB17-Prkdc^SCID^), resulted in gradual and persistent tumor growth: tumor growth was evident on post-inoculation days 20–25 and continued to grow on days 40–50 (Figure 1A). Interestingly, we observed an early onset of itch, prior to the onset of tumor growth: marked scratching behavior began on day 5 (~50 scratching bouts/hour) and peaked on day 20 (>100 scratching bouts/hour) (Figure 1B). Notably, pruritus continued at high levels (>100 scratching bouts/hour) in the late phase (CTCL days 40–60), highlighting the presence of chronic itch (Figure 1B). We compared tumor growth and pruritus in both sexes and found that female mice developed larger tumors in the late phases (*P* < 0.05 on day 40, *P* < 0.01 on days 50 and 60, Supplemental Figure 1, A and B; supplemental material available online with this article; https://doi.org/10.1172/JCI160807DS1). Female mice also showed a tendency toward greater numbers of scratching bouts (Supplemental Figure 1C). Mouse CTCL was also associated with persistent alloknesis, a mechanical itch induced by von Frey filaments (Figure 1C).

As expected, we found that CTCL mice had hyperplasia, an increase in the thickness of the epidermis (Figure 1, D–F). A time-course analysis revealed that this hyperplasia was correlated with the development of itch that had a rapid onset on day 5 and was maintained out to day 60 (Figure 1F). Mast cells play an important role in eliciting itch in part through histamine-dependent mechanisms (45). Toluidine blue staining revealed a rapid and persistent increase in mast cell numbers (Figure 1, G and H). We detected a significant increase on day 5 (*P* < 0.05, Figure 1 H) that was tightly associated with the development of pruritus in this model (Figure 1B).

We further investigated the involvement of additional immune cell types, including macrophages (CD68^+^), neutrophils (Gr-1^+^), and DCs (CD11c^+^) in this model (Figure 1, I–K and Supplemental Figure 2, A–C). A time-course study showed rapid (<5 days) and persistent (>60 days) increases in the number of macrophages (CD68^+^, *P* = 0.0002, Figure 1I and Supplemental Figure 2A), neutrophils (Gr-1^+^, *P* < 0.0001, Figure 1J and Supplemental Figure 2B), and DCs (CD11c^+^, *P* < 0.0001, Figure 1K and Supplemental Figure 2C). Together, these data suggest that CTCL was associated with profound and long-lasting changes in immune cells for innate immunity (e.g., neutrophils and macrophages) and adaptive immunity (e.g., DCs).

We also tested 2 additional malignant cell lines, Hut78 and Hut102, generated from patients with CTCL. Inoculation of either Hut78 or Hut102 tumor cells into the back skin of SCID mice failed to induce tumor growth or itch (Supplemental Figure 3, A–F). Thus, lymphoma and pruritus may be specifically induced by Myla cells.

### CTCL induces time-dependent and nerve injury–associated neurochemical changes in dorsal root ganglion neurons and their central terminals.

**Figure 2:**
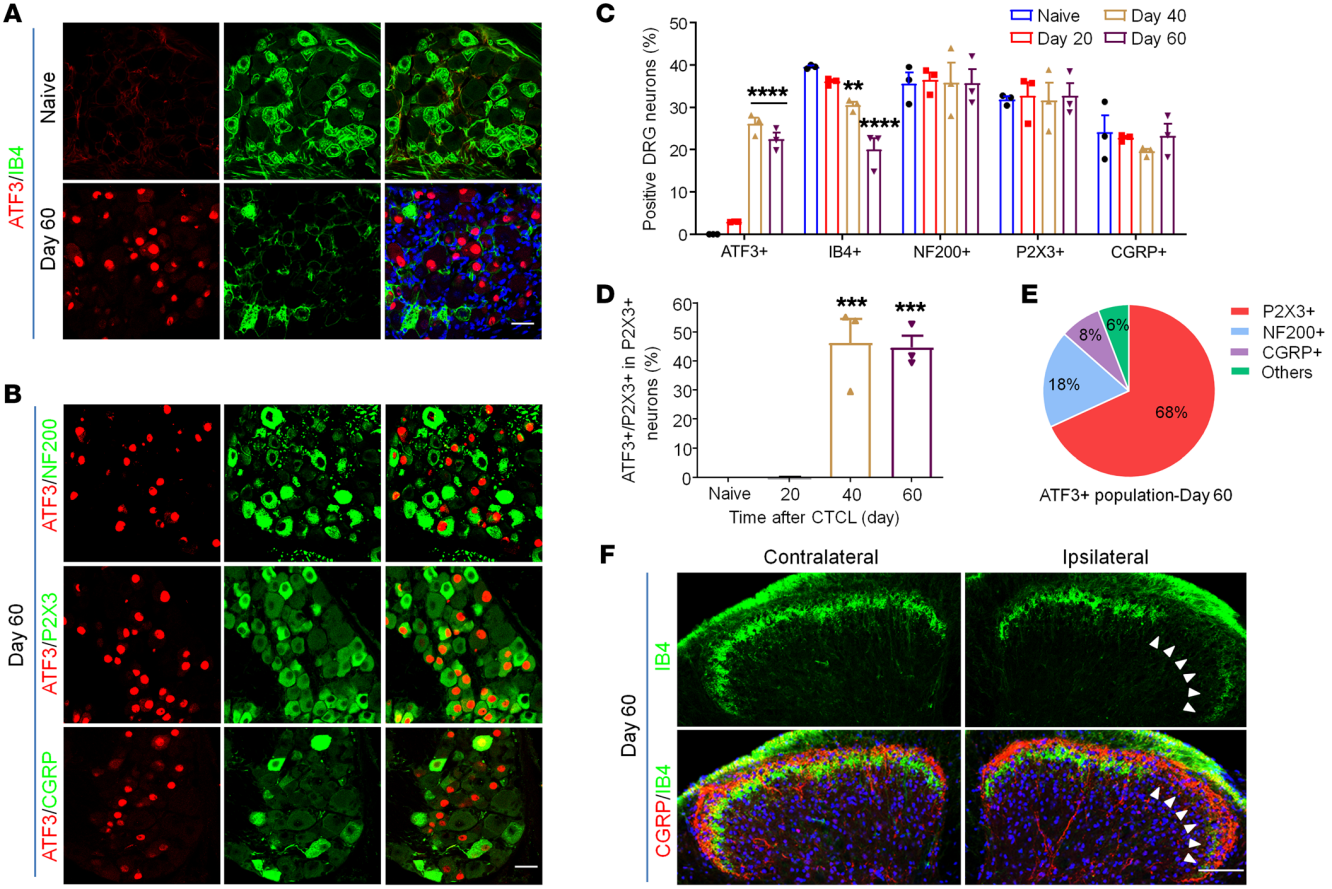
CTCL results in nerve injury in the late phases. (**A**) Double immunostaining for ATF3 and IB4 in cervical DRGs of naive and CTCL mice at day 60. Scale bar: 25 μm. (**B**) Double immunostaining of ATF3 and the neuronal markers NF200, P2X3, and CGRP in cervical DRGs from CTCL mice at day 60. Scale bar: 25 μm. (**C**) Percentage of ATF3^+^, IB4^+^, NF200^+^, P2X3^+^, and CGRP^+^ neurons in the cervical DRG of CTCL mice at different time points. *n* = 3. Two-way ANOVA, *F*_(12,__30)_ = 9.15, *P* < 0.0001. (**D**) Percentage of ATF3 colocalization with P2X3^+^ neurons in the cervical DRG of CTCL mice at different time points. *n* = 3. One-way ANOVA, *F*_(3,__8)_ = 31.89, *P* < 0.0001. (**E**) Pie chart showing ATF3 colocalization with NF200^+^, P2X3^+^, and CGRP^+^ neurons in the cervical DRG from a CTCL mouse on day 60. Data were collected from 3 animals. (**F**) Double immunostaining for CGRP and IB4 in the cervical spinal cord dorsal horn from CTCL mice at day 60. Arrowheads indicate the loss of IB4^+^ primary afferents in the IIi. Scale bar: 100 μm. Data are expressed as the mean ± SEM. One- or 2-way ANOVA with Bonferroni’s post hoc test, **P < 0.01, ***P < 0.001, and ****P < 0.0001 versus the naive group.

Activating transcription factor 3 (ATF3) is a specific marker for nerve injury, as ATF3 is only induced in dorsal root ganglion (DRG) neurons after axonal injury of DRG sensory neurons (46, 47). ATF3 immunostaining showed no or minimum signaling in DRG neurons of naive mice (Figure 2A). We observed a small percentage (3%) of ATF3^+^ neurons on day 20 of CTCL (Supplemental Figure 4A). Strikingly, we found many ATF3^+^ DRG neurons on days 40 and 60 (Figure 2, A and B, and Supplemental Figure 4A). ATF3 was expressed exclusively in the nuclei of DRG neurons (Figure 2, A and B). Quantitative analysis of ATF3^+^ neurons revealed 0%, 3.0%, 26.1%, and 22.5% of DRG neurons expressing ATF3 on days 0, 20, 40, and 60 following CTCL, respectively (Figure 2C and Supplemental Figure 4, A and B). A significant increase in ATF3 did not occur until day 40 (*P* < 0.0001, Figure 2C).

Isolectin B4 (IB4) binds to a subset of nociceptive DRG neurons that do not express neuropeptides (nonpeptidergic), and downregulation of IB4 staining is associated with axonal injury (48, 49). We found that CTCL resulted in significant decreases in IB4^+^ neurons on day 40 (*P* < 0.01) and day 60 (*P* < 0.001) (Figure 2, A and C, and Supplemental Figure 4, A and B). The ATP receptor P2X3 is another marker for nonpeptidergic nociceptors and is downregulated after nerve injury (49). In cervical DRGs (C3–C8), which innervated the tumor-bearing back skin, we did not see a significant reduction in the percentage of P2X3^+^ neurons after CTCL (Figure 2C) but observed significant increases in ATF3 colocalization with P2X3 on days 40 and 60 (*P* < 0.001, Figure 2, B and D). Additionally, we observed high colocalization rates of ATF3 and IB4 on days 40 and 60 (Supplemental Figure 4, B–D).

Calcitonin gene–related peptide (CGRP) is a marker for peptidergic nociceptors of C-fibers and A-fibers; severe nerve injury, such as axotomy of the sciatic nerve, downregulates CGRP expression in DRG neurons (50). CTCL had no significant effects on the CGRP^+^ population at any of the time points examined (Figure 2C). On CTCL day 60, only a small proportion of ATF3^+^ neurons coexpressed CGRP and neurofilament 200 (NF200), a marker for myelinated DRG neurons (Figure 2B). Venn diagram analysis of ATF3^+^ neurons revealed that the majority of these neurons (68%) were P2X3^+^ (Figure 2E), suggesting that CTCL causes axonal injury primarily in nonpeptidergic neurons. Consistently, we found that CTCL caused a substantial reduction of IB4^+^ primary afferents in the inner lamina II (IIi), without affecting the CGRP^+^ primary afferents in the lamina I and outer lamina II (IIo) in the spinal cord dorsal horn on day 60 (Figure 2F). Notably, nonpeptidergic neurons primarily innervate the epidermis where itch originates (1, 51). Double staining for ATF3 and *Trpv1* using RNAscope probes revealed that ATF3 was also expressed by *Trpv1*^+^ neurons on day 60 (Supplemental Figure 4E). Cleaved and active caspase 3 are markers of cell apoptosis (52). On CTCL day 60, we observed some cells that were labeled with cleaved caspase 3, but ATF3 was not coexpressed in these cells (Supplemental Figure 4F). Thus, despite axonal injury, ATF3^+^ neurons may not undergo apoptosis.

### CTCL is characterized by dysregulation of nerve innervations.

**Figure 3:**
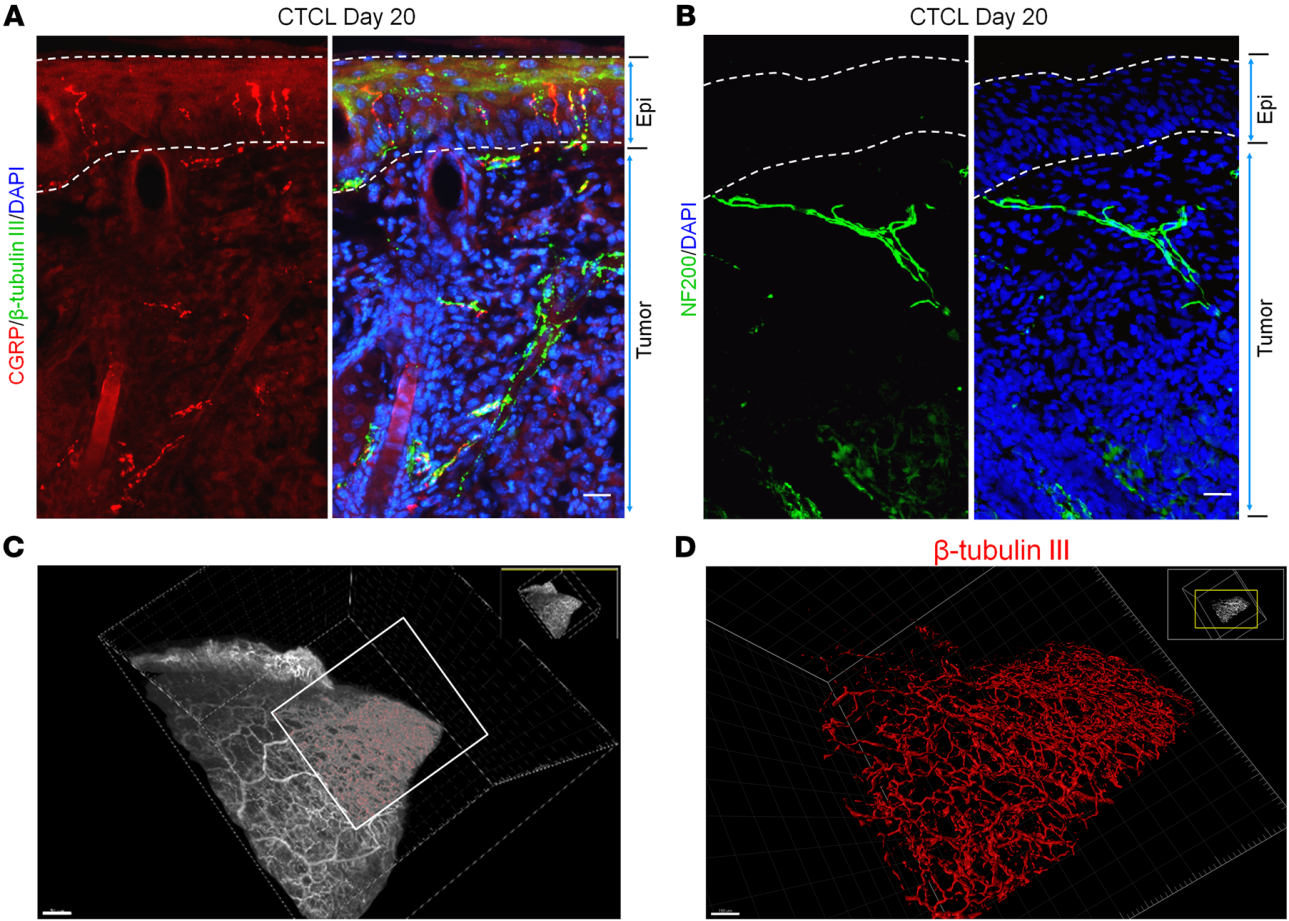
CTCL is associated with nerve innervations in mouse lymphoma. (**A**) Double immunostaining for CGRP and β–tubulin III in the back skin with lymphoma from a CTCL mouse at day 20. Dotted lines show the epidermis. Scale bar: 25 μm. (**B**) Immunostaining for NF200 in the tumor from a CTCL mouse at day 20. Dotted lines show the epidermis. Scale bar: 25 μm. (**C**) 3D reconstruction of innervated nerves in a β–tubulin III–labeled tumor from a CTCL mouse at day 20. Scale bar: 300 μm. (**D**) High-magnification image of the boxed area in **C**. Scale bar: 150 μm.

Since nerve injury occurs in the late phase of CTCL and spontaneous itch peaks on day 20 (Figure 1B), we next investigated nerve innervations in tumor-bearing skin tissue at this time point (Figure 3, A–D, and Supplemental Figure 5). To characterize nerve innervations in the back skin and associated lymphoma, we used several nerve fiber markers: β–tubulin III for all fibers, CGRP for peptidergic afferents, and NF200 for myelinated afferents. We saw innervations of β–tubulin III^+^CGRP^+^ and β–tubulin III^+^ fibers not only in the epidermis and but also within the tumor under the epidermis (Figure 3A). NF200^+^ afferents were also found in the tumor (Figure 3B and Supplemental Figure 5).

To further assess nerve innervations in the lymphoma, we used a clarity method (53). The polyethylene glycol–associated solvent system (PEGASOS) renders all tissues transparent. We performed whole-mount β–tubulin III immunostaining of skin and tumor and then cleared the tissue samples using the PEGASOS immersion method. 3D reconstruction demonstrated that the entire lymphoma was densely innervated by nerve fibers (Figure 3, C and D, Supplemental Video 1).

**Figure 4:**
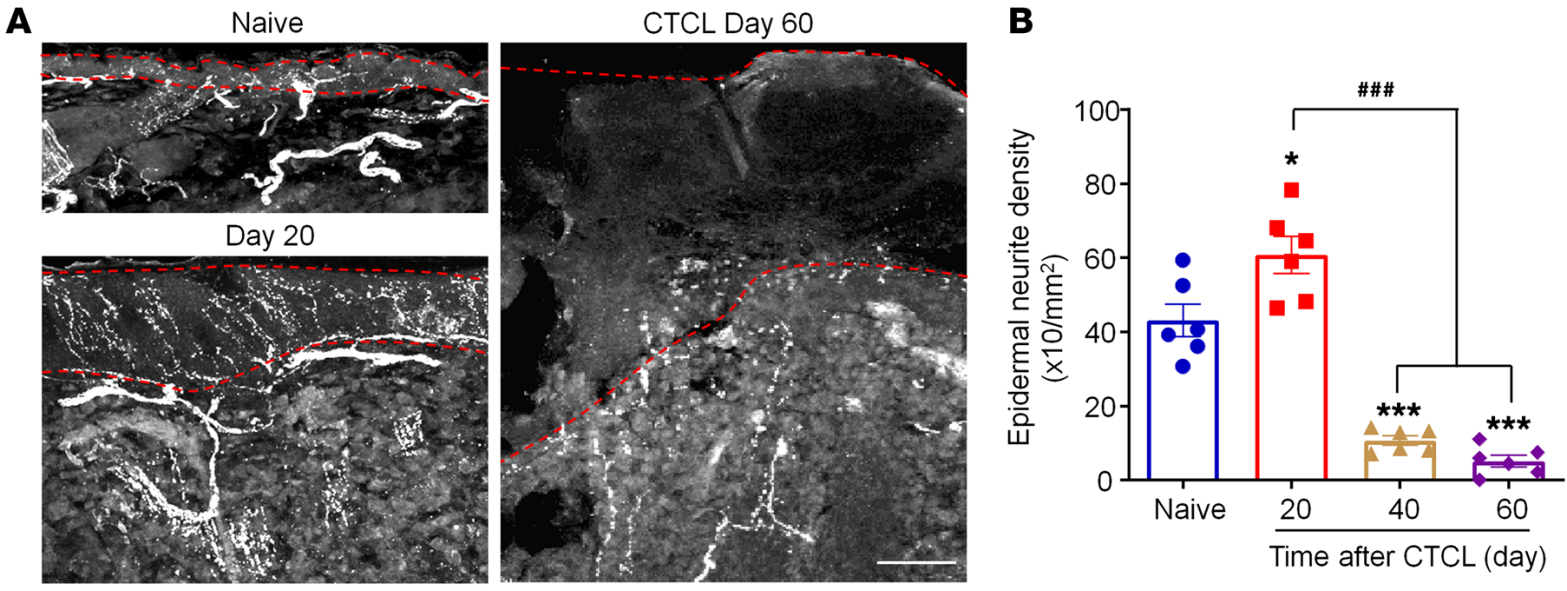
Mouse CTCL is associated with hyperinnervations in the early phase and hypoinnervations in the late phases. (**A**) Immunostaining for β–tubulin III in the tumor from a CTCL mouse at day 0 (naive control), day 20, and day 60. Dotted lines show the epidermis. Scale bar: 40 μm. (**B**) Time course of neurite intensity in the tumor. Data are expressed as the mean ± SEM. *n* = 6. *P < 0.05 and ***P < 0.001 versus the naive group; ^###^*P* < 0.001 versus the CTCL day-20 group, 1-way ANOVA with Bonferroni’s post hoc test, *F*_(3,__20)_ = 58.62, *P* < 0.0001.

Peripheral neuropathy is characterized by loss of epidermal neurite density (END) (54, 55). We quantified END on the basis of β–tubulin III immunostaining (Figure 4, A and B). Intriguingly, we observed a significant increase in END on day 20: 60.78 ± 4.98 × 10/mm^2^ versus 43.05 ± 4.38 × 10/mm^2^ in the control group (*P* < 0.01, Figure 4, A and B). By contrast, late-phase CTCL was associated with substantial reductions of END (Figure 4, A and B), with reductions of approximately 75% on day 40 (*P* < 0.001) and approximately 90 % on day 60 (*P* < 0.001). Notably, lymphoma nerve innervations were still evident on day 60 (Figure 4B). Together, these results suggest that (a) lymphomas are innervated by the peripheral nerves and (b) the epidermis exhibits paradoxical hyper- and hypo-nerve innervations in the early and late phases of CTCL, respectively.

### Nerve blockade suppresses CTCL-induced itch in a time-dependent manner.

**Figure 5:**
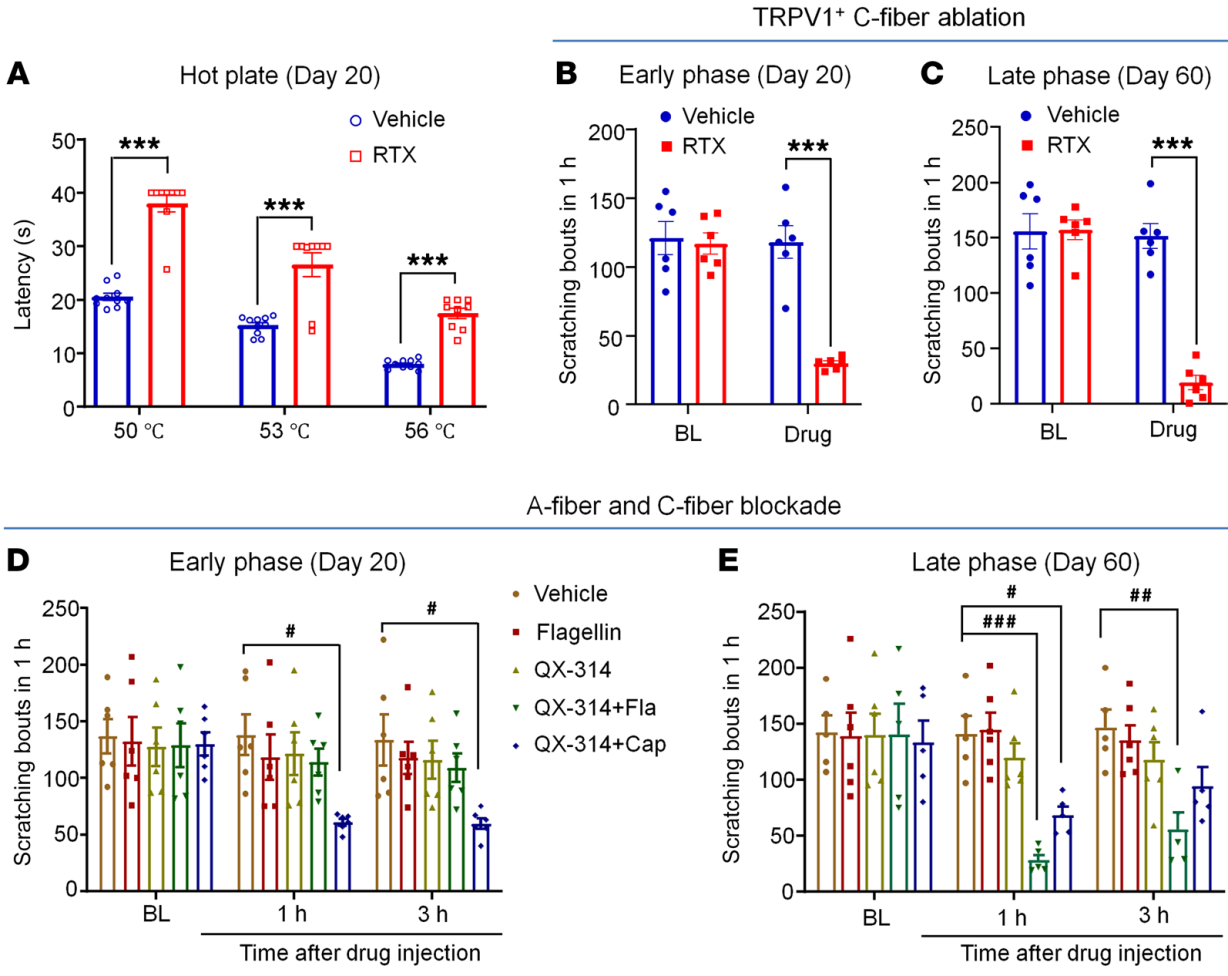
Effects of C-fiber or A-fiber nerve blockade on CTCL-induced pruritus in the early and late phases. (**A**) Response latency in hot-plate tests at 50°C, 53°C, and 56°C in vehicle-treated (*n* = 10) and RTX-treated (*n* = 9) CTCL mice at day 20. *F*_(2,__51)_ = 98.25, *P* < 0.0001. (**B**) CTCL-induced itch in the early phase (day 20) in mice before (baseline [BL])and after the treatment with vehicle (*n* = 6) or RTX (*n* = 6). *F*_(1,__10)_ = 42.93, *P* < 0.0001. (**C**) CTCL-induced itch in mice in the late phase (day 60) before and after the treatment with vehicle (*n* = 6) or RTX (*n* = 6). *F*_(1, 10)_ = 52.77, *P* < 0.0001. (**D**) CTCL-induced itch in the early phase (day 20) in mice before and 1 hour and 3 hours after the treatment with intratumoral injection of vehicle, flagellin (Fla) (1 μg, 30 μL), QX-314 (6 mM, 30 μL), QX-314 (6 mM, 30 μL) plus flagellin (1 μg), or QX-314 (6 mM, 30 μL) plus capsaicin (Cap) (10 μg). *n* = 6/group. *F*_(4,__75)_ = 4.49, *P* = 0.0026. (**E**) CTCL-induced itch in the late phase (day 60) in mice before and 1 hour and 3 hours after intratumoral injection of vehicle, flagellin (1 μg, 30 μL), QX-314 (6 mM, 30 μL), QX-314 (6 mM, 30 μL) plus flagellin (1 μg), or QX-314 (6 mM, 30 μL) plus capsaicin (10 μg). *n* = 6/group. *F*_(4,__22)_ = 4.20, *P* = 0.0112. Data are expressed as the mean ± SEM. Two-way ANOVA with Bonferroni’s post hoc test, ^#^*P* < 0.05, ^##^*P* < 0.01, ^###^*P* < 0.001, and ***P < 0.001.

Transient receptor potential ion channel V1–expressing (TRPV1-expressing) C-fibers are essential for the generation of histamine-dependent and -independent pruritus (1, 6, 56, 57). To ablate and/or inactivate TRPV1^+^ C-fibers, we treated animals bearing CTCL with the ultrapotent TRPV1 agonist resiniferatoxin (RTX) (58). A hot-plate test revealed heat hypoalgesia after the RTX treatment (Figure 5A). Notably, RTX treatment produced a profound inhibition of scratching in both the early phase (CTCL day 20, *P* < 0.001, Figure 5B) and late phase (CTCL day 60, *P* < 0.001, Figure 5C). Thus, TRPV1^+^ afferents were critically required for the development and maintenance of CTCL-induced pruritus.

QX-314, a derivative of lidocaine, is a cell membrane–impermeable sodium channel inhibitor. Notably, QX-314 has selective access to C-fibers by coapplication with capsaicin (59) or to A-fibers (preferentially Aβ fibers) by coapplication with the TLR5 agonist flagellin (60). We used the QX-314–based approach to block C-fibers and A-fibers in both early and late phases. C-fiber blockade with intradermal/intratumoral administration of 6 mM QX-314 plus 10 μg capsaicin significantly reduced scratching on both CTCL day 20 and day 60 (*P* < 0.05, Figure 5, D and E). Interestingly, A-fiber blockade with 6 mM QX314 plus 1 μg flagellin significantly reduced pruritus on CTCL day 60 (*P* < 0.01, Figure 5E), but showed no effect on CTCL day 20 (Figure 5D). These results suggest a distinct contribution of C- and A-fibers to acute and chronic itch after CTCL.

### IL-31 signaling pathway is upregulated and critically contributes to pruritus after CTCL.

**Figure 6:**
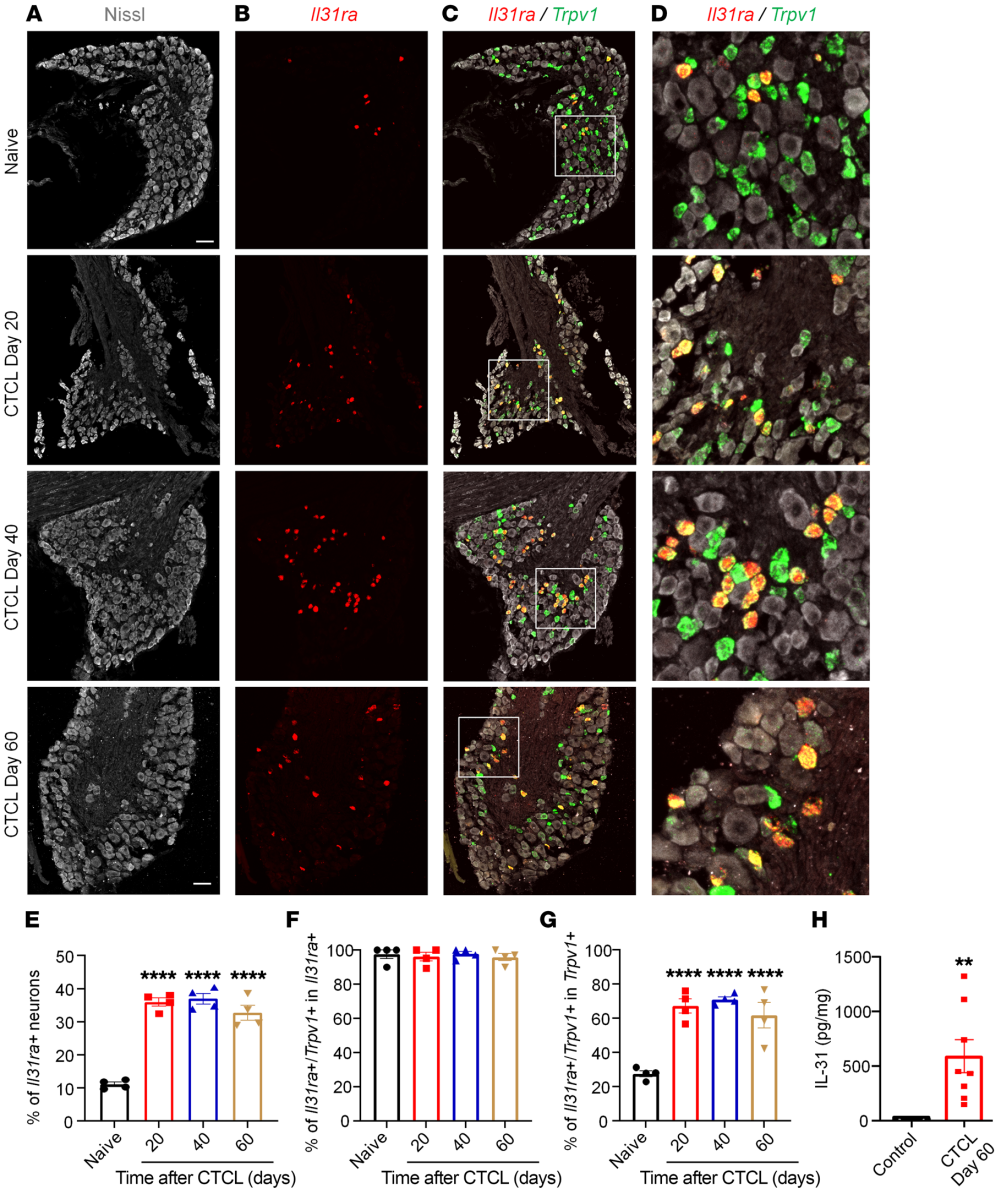
The IL-31 signaling pathway is upregulated and critically contributes to pruritus after CTCL. (**A**–**C**) Triple staining showing Nissl-labeled neurons (**A**), *Il31ra* mRNA (RNAscope, red, **B**), and *Trpv1* mRNA (RNAscope, green, **C**) at different phases of CTCL. Scale bars: 60 μm. (**D**) Enlarged images from the boxed areas in **C**. Original magnification, ×20. Note the increase in DRG neurons expressing *Il31ra* after CTCL. Also note that *Il31ra* is heavily colocalized with *Trpv1* in the same DRG neurons of naive and CTCL mice. (**E**) Quantitative analysis showing the percentage of *Il31ra^+^* DRG neurons in naive and CTCL mice (*n* = 4). *P* < 0.0001 versus naive, 1-way ANOVA, *F*_(3, 12)_= 62.77. (**F** and **G**) Percentage of double-labeled neurons in the *Il31ra* (**F**) and *Trpv1* (**G**) populations. *n* = 4 animals. *P* < 0.0001 versus naive, 1-way ANOVA, *F*_(3,__12)_= 0.1945 (**F**), *F*_(3,__12)_= 20.25 (**G**). (**H**) ELISA results showing low levels of IL-31 in nonmalignant control skin tissue (*n* = 5, with acute Myla cell inoculation) and significantly higher levels of IL-31 in CTCL day-60 lymphoma-bearing skin tissue (*n* = 8). **P < 0.01 and ****P < 0.0001, versus control, unpaired, 2-tailed Student’s *t* test, *t* = 2.929*, df* = 11.

**Figure 7:**
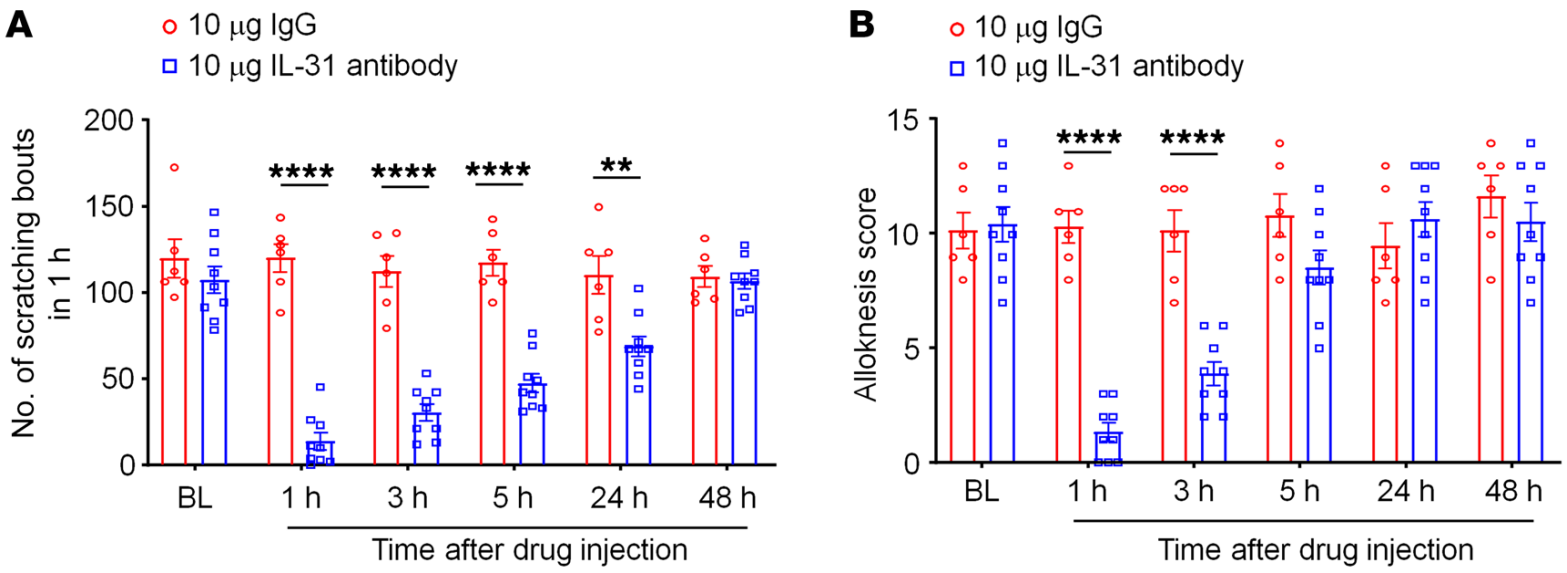
A single intratumoral treatment with IL-31–neutralizing antibody reduces CTCL-induced pruritus. (**A**) Number of scratching bouts in 1 hour. Compared with IgG control antibody, IL-31–neutralizing antibody significantly reduced scratching at 1 hour, 3 hours, 5 hours, and 24 hours. Two-way ANOVA, *P* < 0.0001, *F*_(1,__78)_= 162.9. (**B**) Alloknesis induced by von Frey filament. Compared with IgG control antibody, IL-31–neutralizing antibody significantly reduced CTCL-induced alloknesis at 1 hour and 3 hours. Two-way ANOVA; *P* < 0.0001, *F*_(1,__72)_= 40.09. *n* = 6 for control IgG treatment; *n* = 9 for IL-31 antibody treatment. Behavior was assessed on CTCL day 25. **P < 0.01 and ****P < 0.0001, by Bonferroni’s multiple-comparison test.

IL-31 has been widely implicated in CTCL-associated pruritus in patients (61–63). To investigate the role of IL-31 signaling in CTCL-induced itch in mice, we conducted ISH, ELISA, and behavioral analyses. ISH with RNAscope revealed that a greater number of DRG neurons expressed *Il31ra* mRNAs after CTCL (Figure 6, A and B). Double staining further demonstrated a very high ratio of colocalization of *Il31ra* and *Trpv1* transcripts in naive and CTCL mice (Figure 6, C and D). Quantitative analysis showed that the percentage of *Il31ra*^+^ DRG neurons increased from 10% in naive mice to more than 30% in CTCL mice between 20 and 60 days (*P* < 0.01, vs. naive, Figure 6E). Furthermore, more than 90% of *Il31ra*^+^ neurons expressed *Trpv1* (Figure 6F), and approximately 60% of *Trpv1*^+^ neurons expressed *Il31ra* in CTCL mice (Figure 6G). ELISA results showed low levels of IL-31 in nonmalignant skin tissue but a substantial increase in IL-31 levels in lymphoma tissues (*P* < 0.0001, vs. control, Figure 6H). Thus, both the ligand and receptor for the IL-31 signaling pathway were upregulated in CTCL tissues. Strikingly, intratumoral administration of an IL-31–neutralizing antibody (10 μg, 10 μL) significantly inhibited CTCL-induced scratching at 1 hour (*P* < 0.0001), 3 hours (*P* < 0.0001), 5 hours (*P* < 0.01), and 24 hours (*P* < 0.05) compared with control IgG (Figure 7A). The same treatment also significantly reduced CTCL-induced alloknesis at 1 hour (*P* < 0.0001) and 3 hours (*P* < 0.0001) compared with control IgG (Figure 7B). Together, these data suggest that the IL-31 signaling pathway was upregulated in mouse CTCL and critically regulated pruritus in this model.

### Gabapentin suppresses CTCL-induced itch in a time-dependent manner.

**Figure 8:**
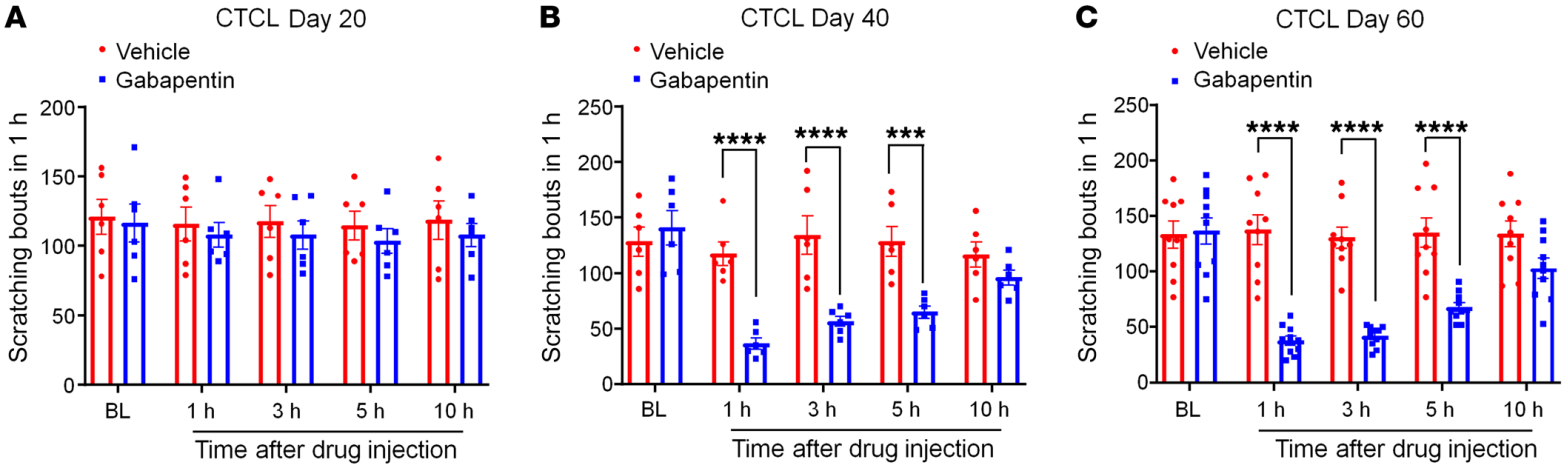
Intrathecal injection of 100 μg gabapentin inhibits neuropathic itch in the late phases. (**A**) Effects of vehicle (*n* = 6) and gabapentin (*n* = 6) on CTCL-induced itch at day 20. *F*_(1,__50)_ = 1.54, *P* = 0.22. (**B**) Effects of vehicle (*n* = 6) and gabapentin (*n* = 6) on CTCL-induced itch at day 40. *F*_(1,__50)_ = 42.94, *P* = 0.0015. (**C**) Effects of vehicle (*n* = 9) and gabapentin (*n* = 10) on CTCL-induced itch at day 60. *F*_(1, 85)_ = 84.61, *P* < 0.0001. Data are expressed as the mean ± SEM. Two-way ANOVA with Bonferroni’s post hoc test; ***P < 0.001 and ****P < 0.0001.

Gabapentin is an anticonvulsant and is widely used to treat neuropathic pain (64–66). Gabapentin (100 μg) was intrathecally (i.t.) injected (66) into CTCL animals at 20, 40, and 60 days, and scratching was assessed 1, 3, 5, and 10 hours after the i.t. injection (Figure 8, A–C). Gabapentin significantly reduced pruritus in the late phases on day 40 (*P* < 0.001, Figure 8B) and day 60 (*P* < 0.0001, Figure 8C), reducing scratching for more than 5 hours (Figure 8, B and C). However, gabapentin failed to affect scratching on day 20 (Figure 8A). Thus, CTCL-induced itch was highly responsive to gabapentin in the late phases.

### CTCL is associated with cognitive decline in early and late phases.

**Figure 9:**
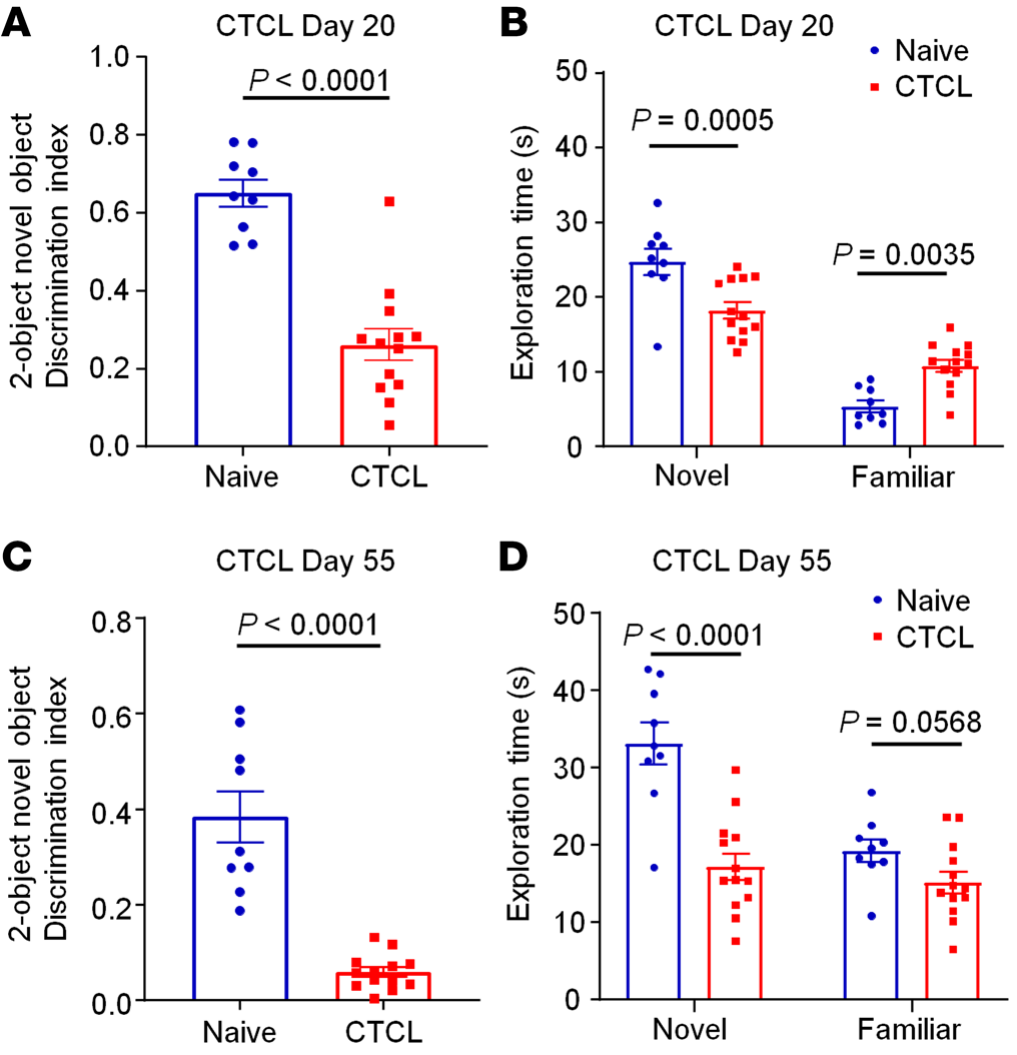
CTCL is associated with impaired cognition in the early and late phases. (**A** and **B**) Two-object NOR discrimination tests were performed to determine the discrimination index (**A**) and exploration time (**B**) for naive mice (*n* = 9) and early-phase CTCL mice (day 20, *n* = 13). (**C** and **D**) Two-object NOR tests to determine the discrimination index (**C**) and exploration time (**D**) for naive mice (*n* = 9) and late-phase CTCL mice (day 55, *n* = 13). Data are expressed as the mean ± SEM. Significance was determined by unpaired, 2-tailed Student’s *t* test.

Chronic pain is associated with cognitive decline and abnormalities in hippocampal functioning (67). We tested the hypothesis that chronic itch is also associated with cognitive deficits by examining cognitive behaviors of mice in early and late phases of CTCL (Figure 9, A–D). Novel object testing showed substantial decreases in the discrimination index on day 20 (*P* < 0.0001, Figure 9A) and day 55 (*P* < 0.0001, Figure 9C). Further analysis of the new object exploration time also revealed significant reductions in exploration time on CTCL day 20 (*P* < 0.01, Figure 9B) and CTCL day 55 (*P* < 0.0001, Figure 9D).

### CTCL-induced synaptic plasticity in cervical spinal cord neurons is suppressed by neuroprotectin D1.

**Figure 10:**
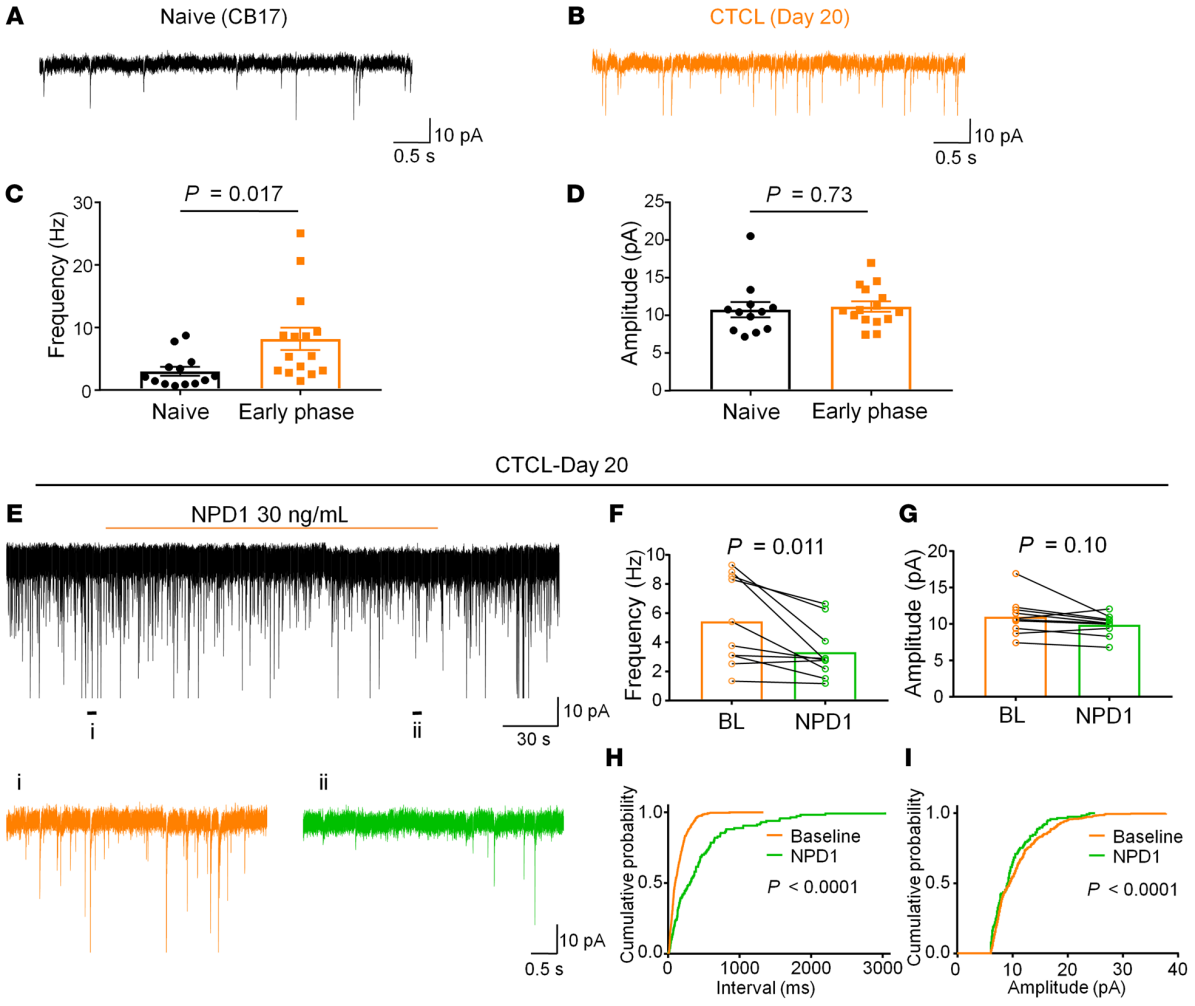
Excitatory synaptic transmission in spinal cord slices from naive and CTCL mice. (**A** and **B**) Representative traces of sEPSCs from spinal dorsal horn neurons from naive CB17 and early-phase CTCL mice. (**C** and **D**) CTCL was associated with significant increase in sEPSC frequency (**C**) but not sEPSC amplitude (**D**). Unpaired, 2-tailed Student’s *t* test, *n* = 12 neurons (naive) and *n* = 15 neurons (CTCL). (**E**–**I**) Inhibition of sEPSCs by perfusion of spinal cord slices with NPD1 (30 ng/mL) in CTCL day-20 mice. (**E**) Representative traces of sEPSCs before and during the perfusion of NPD1 (30 ng/mL). Bottom: Enlarged sEPSC traces on a short time scale. (**F** and **G**) NPD1 significantly reduced sEPSC frequency without affecting sEPSC amplitude. Significance was determined by paired, 2-tailed Student’s *t* test. *n* = 10 neurons/group. (**H** and **I**) Cumulative histograms of the inter-event intervals and amplitudes of sEPSCs before and after NPD1 perfusion. The histograms were examined for 1 minute before and during NPD1 treatment. The inter-event interval was significantly prolonged and the amplitude was significantly decreased by NPD1. Significance was determined by Kolmogorov-Smirnov 2-sample test. Data indicate the mean ± SEM. *n* = 3–4 animals/group.

Spinal cord synaptic plasticity drives the development of chronic pain and chronic itch (22, 68). We used spinal cord slice preparation to assess excitatory and inhibitory synaptic transmission in the cervical spinal cord (C3) that innerves the back skin with tumor inoculation. Patch-clamp recordings in spinal slices from early-phase CTCL mice revealed marked increases in spontaneous excitatory postsynaptic currents (sEPSCs) in lamina II neurons (Figure 10, A and B). We found a significant increase in sEPSC frequency (*P* < 0.05) but not sEPSC amplitude (Figure 10, C and D). We also recorded spontaneous inhibitory postsynaptic currents (sIPSCs) in lamina II neurons of cervical spinal cord neurons and did not see significant changes in sIPSC frequency or amplitude (Supplemental Figure 6, A and B), suggesting that disinhibition (loss of inhibitory synaptic transmission) may not be a major mechanism in this chronic itch model. CTCL further increased the frequency of sEPSCs in spinal cord neurons in the late phases (Supplemental Figure 6, C and D).

Neuroprotectin D1 (NPD1) is a specialized proresolving mediator (SPM), derived from omega-3 polyunsaturated fatty acids (docosahexaenoic acids [DHAs]) and has shown potent analgesic actions in animal models of inflammatory and neuropathic pain (66, 69). Furthermore, i.t. NPD1 effectively suppressed pruritus in the CTCL model (70). Perfusion of spinal cord slices from CTCL mice (day 20) with NPD1 (30 ng/mL) significantly (*P* < 0.05) reduced the sEPSC frequency but not the sEPSC amplitude (Figure 10, E–I). Thus, NPD1 may control chronic itch by normalizing lymphoma-induced synaptic plasticity in the spinal cord.

### CTCL-induced itch requires TLR4 in both phases and both sexes.

**Figure 11:**
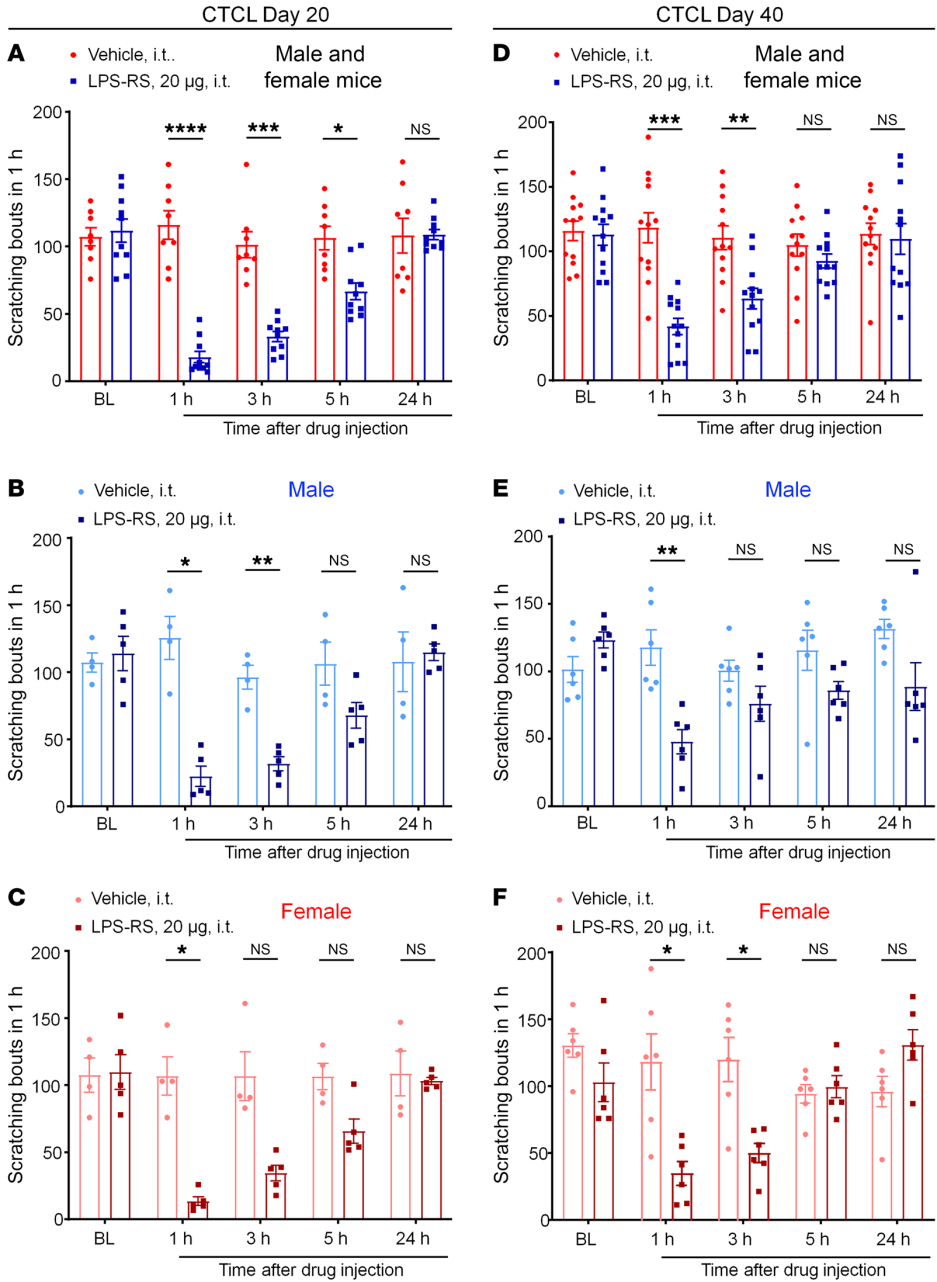
Intrathecal injection of the TLR4 antagonist LPS-RS (20 μg) reduces CTCL-induced itch in early and late phases in males and females. (**A**–**C**) CTCL-induced itch in the early phase (day 20). (**A**) Effects of vehicle (*n* = 8) and LPS-RS (*n* = 10) in male and female mice. *F*_(1,__16)_ = 48.27, *P* < 0.0001. (**B**) Effects of vehicle (*n* = 4) and LPS-RS (*n* = 5) in male mice. *F*_(1,__7)_ = 18.49, *P* = 0.0036. (**C**) Effects of vehicle (*n* = 4) and LPS-RS (*n* = 5) in female mice. *F*_(1,__7)_ = 25.45, *P* = 0.0015. (**D**–**F**) CTCL-induced itch in the late phase (day 40). (**D**) Effects of vehicle (*n* = 12) and LPS-RS (*n* = 12) in male and female mice. *F*_(1,__22)_ = 33.11, *P* < 0.0001. (**E**) Effects of vehicle (*n* = 6) and LPS-RS (*n* = 6) in male mice. *F*_(1,__10)_ = 32.88, *P* = 0.0002. (**F**) Effects of vehicle (*n* = 6) and LPS-RS (*n* = 6) in female mice. *F*_(1,__10)_ = 8.942, *P* = 0.0136). Data are expressed as the mean ± SEM. Two-way ANOVA with Bonferroni’s post hoc test; *P < 0.05, **P < 0.01, ***P < 0.001, and ****P < 0.0001.

TLR4 plays an important role in dry skin–induced persistent itch (68). We examined the involvement of TLR4 in CTCL-induced itch by testing the effects of 20 μg of the TLR4 antagonist LPS *Rhodobacter sphaeroides* (LPS-RS) in both sexes (Figure 11, A–F). Intrathecal LPS produced significant reductions in scratching at 1 hour (*P* < 0.0001), 3 hours (*P* < 0.001), and 5 hours (*P* < 0.05) on CTCL day 20 in animals of both sexes (Figure 11A). TLR4 was shown to regulate inflammatory pain and neuropathic pain in male mice but not female mice (71, 72). To test for sex dimorphism in TLR4 signaling in itch, we separately analyzed the data from male mice (Figure 11B) and female mice (Figure 11C). Interestingly, i.t. LPS-RS suppressed pruritus in both sexes, showing significant reductions in scratching at 1–5 hours in males and females (Figure 11, B and C). Furthermore, LPS-RS reduced CTCL-induced itch on CTCL day 40 among mice of both sexes (Figure 11D), males only (Figure 11E), and females only (Figure 11F). Together, these data suggest that spinal cord TLR4 signaling is critically required for the lymphoma-induced itch in both sexes. In contrast, intratumoral administration of LPS-RS, at doses of 20 μg and 50 μg, failed to suppress CTCL-induced itch on CTCL days 27 and 37 (Supplemental Figure 7, A and B). These results suggest that TLR4 promotes pruritus after CTCL through central modulation.

## Discussion

Peripheral neuropathy is the most common etiology of neuropathic itch (37). Unfortunately, there is lack of relevant animal models to study the mechanisms and treatments of this detrimental disease. The current animal models of neuropathy, such as nerve injury of the sciatic nerve and its branches (73–75), diabetic neuropathy (55), and chemotherapy-induced peripheral neuropathy (76), do not feature spontaneous itch, although mechanical itch (alloknesis) could develop in diabetic mice treated with streptozotocin (77). Several mouse genetic models of neuropathic itch have been reported. Mice lacking vesicular glutamate transporter type 2 (VGLUT2) in primary afferents show reduced responses to noxious mechanical, thermal, and chemical inputs. However, these mutant mice also exhibit spontaneous itch behavior and elevated responses to pruritogens, providing an example of pain suppression of itch (34). Mutant mice with loss of GABAergic interneurons, due to a deletion of the transcription factor Bhlhb5, display severe and nonremitting chronic itch (14). Targeted transplantation of precursor GABAergic neurons into the spinal cord result in a dramatic reduction of scratching and resolution of skin lesions (40). Scratch-induced skin lesions further exacerbate pruritus in these genetic models (14, 34). Although these models do mimic molecular and cellular mechanisms of neuropathic itch, they do not recapitulate human disease conditions.

In this study, we characterized a mouse model of neuropathic itch induced by intradermal inoculation of CTCL on the back skin. We provide several lines of histochemical, pharmacological, and behavioral evidence for time-dependent development of neuropathic itch in a mouse CTCL model. First, our analysis of END revealed an initial increase in END on day 20, which was correlated with increased thickness of the epidermis (hyperplasia) and robust scratching, as demonstrated in dry skin injury, toxic contact dermatitis, and allergic contact dermatitis models (27–31). Thus, the early phase of CTCL represents a dermatitis model of itch. Second, late phase of CTCL (days 40–60) was associated with a substantial (>75%) reduction in END. The peripheral neuropathy was also validated by ATF3 expression in lymphoma-innervating cervical DRGs in the late phase (days 40–60) but not in the early phase (day 20). Loss of IB4 staining was further observed in the spinal cords of CTCL mice on day 60. Third, C-fiber blockade suppressed the CTCL-induced pruritus in both early and late phases, whereas A-fiber blockade alleviated itch only in the late phase. Consistently, QX-314/flagellin-based A-fiber blockade effectively inhibited tactile allodynia in neuropathic pain after chemotherapy (78) and suppressed mechanical itch and spontaneous itch after skin injury (18). Importantly, we found that gabapentin, a commonly used treatment for neuropathic pain, specifically reduced pruritus in the late phase (days 40 and 60) but not in the early phase (day 20).

Abnormal itching may serve as the first sign of an underlying malignancy; and malignancy-associated itch may result from a tumor’s local effects or from a systemic reaction to circulating factors and itch mediators produced by a tumor (63), such as miR-711 (44). Lymphoproliferative malignancies are the most common reasons for paraneoplastic itch, and the severity of itch correlates with disease progression (63). Although melanoma is the most common skin cancer, malignancy-associated pruritus mainly results from nonmelanoma skin cancers. There are over 1 million new cases of malignancy-associated pruritus every year due to nonmelanoma skin cancers (63). CTCLs are the most frequent primary skin lymphomas and cause intractable pruritus, especially in advanced stages (42, 63). One retrospective study of 486 patients with CTCL revealed that 66% of these patients experienced itching (79, 80). Currently, there are no standardized procedures for treating pruritus in CTCL (37, 43). While there are multiple clinical subtypes of CTCL, this study was focused on MF, because (a) MF is the most common form of CTCL (81); (b) the CD4^+^ Myla cell line used in this study was from a patient with MF; and (c) itch severely impairs the quality of life of patients with MF (82). We speculate that the pruritus in these patients could be improved with topical and systemic antiinflammatory agents or retinoids in early phases, before the development of neuropathy. However, refractory itching could benefit from treatment that can target central mechanisms or both peripheral and central mechanisms. For example, the neurokinin 1 receptor antagonist aprepitant was shown to produce rapid and substantial inhibition of pruritus in 3 patients with CTCL (Sézary syndrome and MF) (83). This may work via inhibition of central sensitization in spinal dorsal horn neurons and/or inhibition of mast cells.

One of the most interesting findings of this study is the dramatic upregulation of the IL-31 signaling pathway in this model. Recent studies have implicated IL-31 in pruritus in patients with CTCL (63). Epidermal IL-31 levels correlated with itch severity in patients with CTCL with moderate-to-severe pruritus. IL-31 is elevated in the epidermis and dermal infiltrate, whereas the IL-31 receptor (IL-31RA) and its coreceptor OSMRβ are elevated only in the epidermis (61). Furthermore, IL-31 serum levels are higher in CTCL patients than in controls (62). We provided several lines of evidence for a critical contribution of this signaling pathway in the mouse CTCL model: (a) *Il31ra* was upregulated in *Trpv1^+^* neurons in CTCL mice on days 20, 40, and 60; (b) IL-31 levels were significantly increased in lymphomas; and (c) a single intratumoral injection of IL-31–neutralizing antibody suppressed CTCL-induced pruritus for more than 24 hours. Mechanistically, activation of IL-31RA has been shown to sensitize TRPV1 and TRPA1 ion channels for pruritus (84, 85).

Several miRNAs including miR-711 are upregulated in skin biopsies from patients with lymphoma (42, 86). We recently identified miR-711 as a novel itch mediator in the CTCL model, which is associated with increased local and serum levels of hsa–miR-711, released from inoculated human cancer cells (44). miR-711 can directly bind the ion channel TRPA1 to elicit TRPA1-dependent itch, whereas neutralization of miR-711 is sufficient to inhibit pruritus after CTCL (44). Spinal opioid receptors are involved in cancer-induced itch, as i.t. administration of naloxone is highly effective in reducing itch after CTCL (19). Spinal glial cells not only regulate neuropathic pain but also contribute to chronic itch including neuropathic itch in lymphoma (87, 88).

Increasing evidence has demonstrated sex dimorphism in pain, involving different cell types, such as microglia, macrophages, T cells, and primary sensory neurons (88–92). In particular, TLR4 regulates microglial signaling and inflammatory and neuropathic pain in male animals (71, 93). TLRs play important roles in both pain and itch (94). However, sex dimorphism in itch has not been carefully investigated. Our data demonstrated that i.t. administration of TLR4 antagonist was highly effective in reducing pruritus in both early and late phases, whereas intratumoral injection of the same antagonist was not effective. Surprisingly, we did not see any sex differences in TLR4-mediated itch, as i.t. LPS-RS equally suppressed itch in mice of both sexes and in both phases of CTCL. Thus, our finding highlighted distinct sex dimorphism in pain and itch.

SPMs, such as resolvins, protectins, and maresins, are derived omega-3 polyunsaturated fatty acids and display analgesic potency in animal models of inflammatory pain and neuropathic pain (66, 95). NPD1 was shown to potently inhibit inflammatory pain and neuropathic pain, as well as spinal cord synaptic plasticity in chronic pain (66, 69). Intrathecal NPD1 also effectively suppressed chronic itch in CTCL (70). Like neuropathic pain, neuropathic itch was also associated with spinal cord synaptic plasticity, as indicated by the dramatic increase in excitatory synaptic transmission (EPSC). Strikingly, this plastic change is reversed by spinal slice perfusion of NPD1. This result indicates that neuropathic pain and neuropathic itch share the common spinal cord mechanisms and that SPMs may be potential therapeutics for both neuropathic pain and neuropathic itch.

In summary, we have characterized a mouse CTCL model of neuropathic and cancer itch that can be used for testing mechanisms and treatments of lymphoma-associated pruritus. Notably, the current animal models of skin damage only show spontaneous itch from several days to several weeks, limiting their translational relevance to chronic neuropathic itch. The CTCL model exhibits remarkable and persistent itch for 2 months, associated with progressive changes in tumor growth and nerve innervations; i.e., hyperinnervations in the early phase and hypoinnervations and neuropathy in the late phase, as well as innervations inside lymphomas in mouse and human samples, allowing for the testing of distinct mechanisms of itch at different times. Approximately, neuropathic itch accounts for 8%–19% of patients affected by chronic pruritus with high severe intensity (8 on a numerical scale of 0 to 10). Itch-causing injury most commonly occurs within the peripheral nervous system but much less commonly within the CNS. The best treatment options for neuropathic itch include anticonvulsants, topical anesthetics, and capsaicin (37). We believe our animal study of CTCL will prompt mechanistic studies on neuropathic itch, as well as a clinical study on CTCL-induced neuropathy, which will lead to new treatment options for the disease. There are several limitations in this study. First, the current CTCL model is established in immune-deficient mice, which makes it difficult to generate this model in transgenic mice. Future studies will consider knockdown or pharmacological approaches to test the contributions of key pruriceptors, such as MrgprA3 and MrgprD (2, 4), natriuretic polypeptide b receptor (9), and gastrin-releasing peptide receptor (8) in the CTCL model. Second, while our data strongly support the involvement of peptidergic neurons (IL-31RA*^+^*TRPV1*^+^*) in CTCL-associated pruritus, we do not exclude the role of nonpeptidergic neurons in which ATF3*^+^* is expressed. Third, A-fiber blockade is effective in reducing itch in the late phase, and lymphomas are innervated by neurofilament*^+^* A-fibers. It remains to be investigated how A-fibers regulate chronic itch in CTCL, and, importantly, whether human lymphomas are innervated by IL-31R^+^ nerve fibers. Finally, our CTCL model is a mixture of both inflammatory and neuropathic components during disease progression. Future studies are warranted to test different effects of antiinflammatory treatments in the early and late phases of CTCL.

## Methods

### Animals.

Immune-deficient mice (NOD.CB17-Prkdc^SCID^, stock no. 001303) were purchased from The Jackson Laboratory and used to generate the lymphoma model. Adult mice (8–20 weeks of age) of both sexes were used for behavioral studies, unless the sex is specified in the figure legends. Mice were group-housed in Duke University animal facilities on a 12-hour light/12-hour dark cycle at 22°C ± 1°C with free access to food and water. No statistical method was used to predetermine sample size. No randomization was applied to the animal experiments. Sample sizes were chosen on the basis of our previous studies on similar tests (19, 44).

### Mouse CTCL Xenograft model of chronic itch.

We developed a murine xenograft model of CTCL using immune-deficient mice (NOD.CB17-Prkdc^SCID^). The CD4^+^ Myla cell line was generated from a patient with mycosis fungoides and purchased from MilliporeSigma (catalog 95051032). For controls, we purchased another 2 lymphoma cell lines from ATCC: Hut78 (ATCC, catalog CRM-TIB-161, generated from a patient with Sézary syndrome) and Hut 102 (ATCC, catalog TIB-162.1, generated from a patient with MF lymphoma). In CB17 mice, CTCL was generated via an intradermal injection of CD4^+^ Myla cells (1 × 10^5^ cells/μL, 100 μL) into the nape of the neck (44). Tumor growth, measured by tumor volume, was assessed for 60 days.

### Drugs and drug injection.

Gabapentin (catalog 60142-96-3), capsaicin (catalog 404-86-4), QX-314 (catalog 21306-56-9), and RTX (catalog R-6712) were purchased from LC Laboratories. LPS-RS (catalog tlrl-rslps) and flagellin (catalog tlrl-pbsfla) were purchases from InvivoGen. To block different types of afferent fibers in the tumor, we conducted multiple site intratumoral injections to deliver flagellin, QX-314, flagellin plus QX-314, and capsaicin plus QX-314, as previously reported (44). To test the role of IL-31 in CTCL-induced itch, IL-31–neutralizing antibody (Thermo Fisher Scientific, catalog 701082) was administrated via intratumoral injections. RTX was administrated following our previous protocol (6). Briefly, RTX was given s.c. for 3 consecutive days with accumulating doses (30, 70, and 100 μg/kg), and animals were subjected to hot-plate tests to validate the treatment efficacy. Gabapentin or LPS-RS was administrated by i.t. injection. LPS-RS was also given by intratumoral injection. For i.t. injection, spinal cord puncture was made by a Hamilton microsyringe (Hamilton) with a 30 gauge needle between the L5 and L6 levels.

### IHC and quantification.

IHC was performed on naive and CTCL animals of both sexes. At the different time points of CTCL, the animals were anesthetized with isoflurane and perfused through the left ventricle first with PBS and then with 4% formaldehyde. After perfusion, skin with tumor, DRG (C3–C8), and spinal cord (C3–C8) tissues were collected and postfixed in 4% formaldehyde at 4°C for 2 hours. Tissues were then placed in a solution of 30% sucrose in PBS at 4°C and dehydrated overnight. Tissues were mounted with optimal cutting temperature medium (Tissue-Tek) and then cut with a cryostat (Leica) at a thickness of 14 μm and thaw-mounted onto Superfrost Plus slides (VWR). The sections were then blocked with blocking buffer (5% donkey serum and 0.1% Triton X-100 in PBS) for 1 hour at room temperature. Afterwards, primary antibodies were diluted in 1% BSA and 0.2% Triton X-100; the sections were then incubated with the primary antibodies overnight at 4°C. The primary antibodies included anti-CGRP antibody (goat, 1:400, Bio-Rad, catalog 1720-9007), anti-ATF3 antibody (mouse, 1:400, Santa Cruz Biotechnology, catalog sc-81189), anti-NF200 antibody (rabbit, 1:5,000, Abcam, catalog ab8135), anti-P2X3 antibody (mouse, 1:500, Neuromics, catalog Gp10108), anti–β–tubulin III antibody (mouse, 1:500, R&D Systems, catalog MAB1195), anti-CD68 antibody (rat, 1:500, BioLegend, catalog 137011), anti–Gr-1 antibody (rat, 1:500, BioLegend, catalog 127623), anti-CD11c antibody (Armenian hamster, 1:500, BD Pharmingen, catalog 550283), cleaved caspase 3 antibody (rabbit, 1:400, Cell Signaling Technology, catalog 9661), and anti–IB4–Alexa Fluor 488 conjugate (Invitrogen, Thermo Fisher Scientific, 1:1,000, catalog 121411). For double staining of DRG and spinal cord sections, 2 primary antibodies from different species were mixed. Following incubation, the sections were washed with PBS and incubated with secondary antibodies (1:500, Jackson ImmunoResearch) for 1–2 hours at room temperature. The secondary antibodies included Cy3-donkey anti-goat (catalog 705-165-003), Cy3-goat anti-mouse (catalog 115-165-003), FITC-donkey anti–rat (catalog 712-097-003), Alexa Fluor 488–donkey anti-mouse (catalog 715-545-151), and Cy3-goat anti–American hamster (catalog 127-165-160). Two secondary antibodies were also mixed for double staining. After washing with PBS, the stained sections were mounted on a coverslip with ProLong Gold mounting medium (Life Technologies, Thermo Fisher Scientific) and allowed to dry overnight at room temperature. Images were obtained using a Leica Confocal Microscope (SP5 Inverted confocal-LSRC, Leica Microsystems) at the Duke University Microscope Center. The number of double-positive cells in 4 skin sections per animal was quantified in a blinded manner and calculated as the number of cells per square millimeters of tissue.

### Whole-mount immunohistochemical staining and imaging of skin and tumor tissues with clarity.

Whole-mount immunohistochemical staining and PEGASOS tissue clearing were performed as previously described (53). In brief, mice were transcardially perfused with 50 mL heparin-PBS (10 U/mL heparin sodium in 0.01 M PBS) and 20 mL 4% paraformaldehyde (PFA) (4% PFA in 0.01 M PBS, pH 7.4), 20 days after intradermal inoculation of CTCL. The skin and associated tumor tissues were dissected and fixed in 4% PFA overnight at room temperature and then subjected to decolorization with 25% Quadrol solution (MilliporeSigma, catalog 122262) for 2 days at 37°C to remove hemoglobin under constant shaking. After washing with the PBS solution for 30 minutes, the samples were immersed in blocking solution composed of 10% dimethyl sulfoxide (MilliporeSigma, catalog 276855), 0.5% IgePal 630 (MilliporeSigma, catalog 18896), and 1× casein buffer (Vector, catalog SP-5020) in 1 mL 0.01 M PBS overnight at room temperature. Samples were then stained with the primary antibody (β–tubulin III, Thermo Fisher Scientific, catalog MA1-118) diluted with the blocking solution (1:200) for 3 days at 4°C on a shaker. After that, samples were washed with PBS at room temperature for 1 day and then stained with the secondary antibodies diluted with the blocking solution for another 3 days at 4°C on a shaker. PBS washing of the samples was done for 8 hours. Following that, serial delipidation was performed at 37°C under constant shaking for 6 hours in each of the following solutions: 30% tert-butanol (tB) (MilliporeSigma, catalog 471712), 50% tB, and 70% tB. Next, samples were dehydrated in tB-PEG solution composed of 70% tB, 27% (v/v) poly(ethylene glycol) methyl ether methacrylate average Mn500 (PEG MMA500, MilliporeSigma, catalog 447943), and 3% (w/v) Quadrol at 37°C. After final clearing with the benzyl benzoate–PEG (BB-PEG) clearing solution composed of 75% (v/v) BB (MilliporeSigma, catalog B6630), 22% (v/v) PEG-MMA500, and 3% (v/v) Quadrol, samples were maintained in the clearing solution for imaging. Whole-tumor tissue fluorescence images were acquired with a LaVision BioTec Ultramicroscope II (Andor Neo) equipped with 6 fixed lightsheet-generating lenses, a complementary metal oxide semiconductor (CMOS) camera at the University of North Carolina at Chapel Hill Microscopy Services Laboratory, and a ×2/0.5 NA objective (MVPLAPO) covered with a 6 mm working distance dipping cap. Version v144 of the Imspector Microscope Controller software supported by LaVision BioTec was used. All raw image data were collected in 8 bit TIFF format. A 3D rendering of the image stacks was made with Imaris 9.0 software (Bitplane).

### RNAscope ISH in mouse DRGs.

DRG sections from naive and CTCL mice at 20, 40, and 60 days were prepared as described for IHC. ISH was performed using the RNAscope system (Advanced Cell Diagnostics) in accordance with the manufacturer’s instructions. We used a protocol designed for the Multiplex Fluorescent Kit version 2 and specific probes directed against murine *Il31ra* (catalog 418411), *Trpv1* (catalog 313331-C3), and *Atf3* (catalog 426891). After completion of the RNAscope protocol, the DRG sections were counterstained with Nissl/NeuroTracer-640 (1:200, Thermo Fisher Scientific, N21483) for 1 hour at room temperature. Sections were mounted with DAPI Fluoromount-G mounting medium (MilliporeSigma). Images were captured with a Zeiss LCM 880 confocal microscope. Quantification was performed in Nissl*^+^* neurons with DAPI*^+^* nucleoli in DRG sections, and neurons with more than 5 puncta (labeled with RNAscope probes) were counted as positive.

### IL-31 ELISA.

IL-31 ELISA kits were purchased from Abcam (catalog ab119546). CTCL with associated skin tissue was collected on day 60. The controls were skin tissues with acute Myla cell inoculation (1 hour). The skin and tumor tissues were homogenized in a lysis buffer containing protease and phosphatase inhibitors (MilliporeSigma, RIPA buffer, catalog R0278), and 50 μg lysed proteins were used for each test well. ELISAs were conducted according to the manufacturer’s instructions, and the standard curve was included for each experiment.

### Behavioral assessment for scratching (pruritus).

Mice were shaved on the nape after a brief anesthesia with isoflurane. Mice were habituated in small plastic chambers (14 × 18 × 12 cm) daily for 2 days before the experiments. The room temperature and humidity remained stable for all the experiments. Mice were then briefly removed from the chamber and given an intradermal or i.t. injection of reagents, with the concentration and volume indicated in the figure legends. After the injection, the number of scratches in 60 minutes was counted. A scratch was counted when a mouse lifted its hind paw to scratch the shaved region and returned the paw to the floor or placed it near its mouth. Scratching was filmed for the time-course study using a Sony HDR-CX610 camera. The video was subsequently played back offline, and the numbers of scratches and wipes were quantified in a blinded manner.

### Alloknesis assay.

Alloknesis was evaluated at different time points for the CTCL models and before and after administration of the IL-31 antibody. Alloknesis in CTCL mice was induced by an innocuous von Frey filament (bending force of 0.07 g) applied to the periphery of the tumor. A scratching bout directed to the site of mechanical stimulation was considered as a positive response. The alloknesis score was determined by calculating the total number of scratches elicited by 5 mechanical stimuli.

### Novel object recognition test.

Mice were habituated in a 30 × 30 × 30 cm square arena (TAP plastics) on the first day. On the following day, 2 identical objects were placed in 2 different corners of the arena, and mice were placed in the arena for a 10-minute exploration. Then, the mice were returned to their home cages for a 30-minute retention interval, and 1 of 2 identical objects was replaced by a new object for the novel object recognition (NOR) test. After the retention interval, the mice were placed back into the testing arena for a 5-minute exploration. Touching an object with its nose or focusing attention on the object at less than 1 cm were defined as a valid exploration by the mouse. Turning around, climbing, or sitting on the object were considered invalid. The discrimination index was calculated as follows: (novel – familiar/novel + familiar) × 100%. Animal behaviors were recorded by a Sony HDR-CX1000 camera and analyzed by a researcher who was blinded to the testing conditions. Data were excluded if the total exploration time was less than 10 seconds.

### Patch-clamp recordings in spinal cord slices from naive and CTCL mice.

CB17 naive and CTCL mice were anesthetized with urethane (1.5–2.0 g/kg, i.p.). After a dorsal laminectomy, the cervical segment of the spinal cord was removed and placed into the preoxygenated, ice-cold cutting solution (240 mM sucrose, 25 mM NaHCO_3_, 2.5 mM KCl, 1.25 mM NaH_2_PO_4_, 0.5 mM CaCl_2_, 3.5 mM MgCl_2_). The mice were then immediately euthanized by decapitation. After the arachnoid membrane was removed, the spinal cord was placed in an agar block and mounted on a metal stage. A transverse slice (400 μm thick) was cut on a vibrating microslicer (VT1200s, Leica). The slices were incubated for 30 minutes in artificial cerebrospinal fluid (ACSF) equilibrated with 95% O_2_ and 5% CO_2_ gas mixture. The ACSF contained: 126 mM NaCl, 3 mM KCl, 2.5 mM CaCl_2_, 1.3 mM MgCl_2_, 1.25 mM NaH_2_PO_4_, 26 mM NaHCO_3_, and 11 mM d-glucose. Spinal cord slices were placed in a recording chamber and perfused at a flow rate of approximately 2 mL/min, with the ACSF equilibrated with a 95% O_2_ and 5% CO_2_ gas mixture and maintained at room temperature. Whole-cell patch-clamp recordings were made from substantia gelatinosa (SG) neurons with patch pipette electrodes that had a resistance of 5–8 MΩ. Lamina II were identified as a translucent band under a microscope (BX51WIF; Olympus) with light transmitted from below (69). All experiments were performed in voltage-clamp mode. The holding potential was set at –70 mV when recording EPSCs and 0 mV when recording IPSCs, respectively. The patch pipette solution for EPSC recordings contained: 135 mM potassium gluconate, 0.5 mM CaCl_2_, 2 mM MgCL_2_, 5 mM KCl, 5 mM EGTA, 5 mM HEPES, and 5 mM ATP-Mg salt, and the patch pipette solution for IPSC recordings contained: 110 mM CsSO_4_, 0.5 mM CaCl_2_, 2 mM MgCl_2_, 5 mM TEA, 5 mM EGTA, 5 mM HEPES, and 5 mM ATP-Mg salt. NPD1 was dissolved in ACSF without alteration of the perfusion rate and bath applied to spinal cord slices by gravity perfusion via a 3-way stopcock. Signals were amplified using an Axopatch 700B amplifier (Molecular Devices) and were filtered at 2 kHz and digitized at 5 kHz. Data were collected and analyzed using pClamp 10.3 software (Molecular Devices). EPSCs and IPSCs were analyzed using Minianalysis, version 6.0.3 (Synaptosoft). In all cases, *n* refers to the number of recorded neurons, which were collected from 3–4 animals for different experiments.

### Statistics.

All data are expressed as the mean ± SEM. Statistical analyses were completed with GraphPad Prism 9.3.0 (GraphPad Software). Biochemical and behavioral data were analyzed using a 2-tailed Student’s *t* test (2 groups) or a 1- or 2-way ANOVA followed by Bonferroni’s post hoc test. The criterion for statistical significance was a *P* value of less than 0.05.

### Study approval.

All animal procedures were approved by the IACUC of Duke University. Animal experiments were conducted in accordance with the NIH’s *Guide for the Care and Use of Laboratory Animals* (National Academies Press, 2011).

## Author contributions

OC, Q He, Q Han, and RRJ developed the project. OC, Q He, Q Han, KF, YG, and MO conducted experiments and data analyses. OC and RRJ prepared the manuscript, and all co-authors edited the manuscript.

## Supplementary Material

Supplemental data

Supplemental video 1

## Version 1 12/15/2022

In-Press Preview

## Version 2 02/15/2023

Electronic publication
