# Supplementary material for: Mechanisms and treatments of neuropathic itch in a mouse model of lymphoma
Source: J Clin Invest. 2023 Feb 15;133(4):e160807. doi: 10.1172/JCI160807 (PMC9927942; doi:10.1172/JCI160807)
Supplement: Supplemental data [file jci-133-160807-s210.pdf]

Mechanisms and treatments of neuropathic itch in a mouse model of lymphoma

Ouyang Chen, Qianru He, Qingjian Han, Kenta Furutani, Yun Gu, Maddie Olexa, Maria and Ru-Rong Ji

Supplemental figures (7)

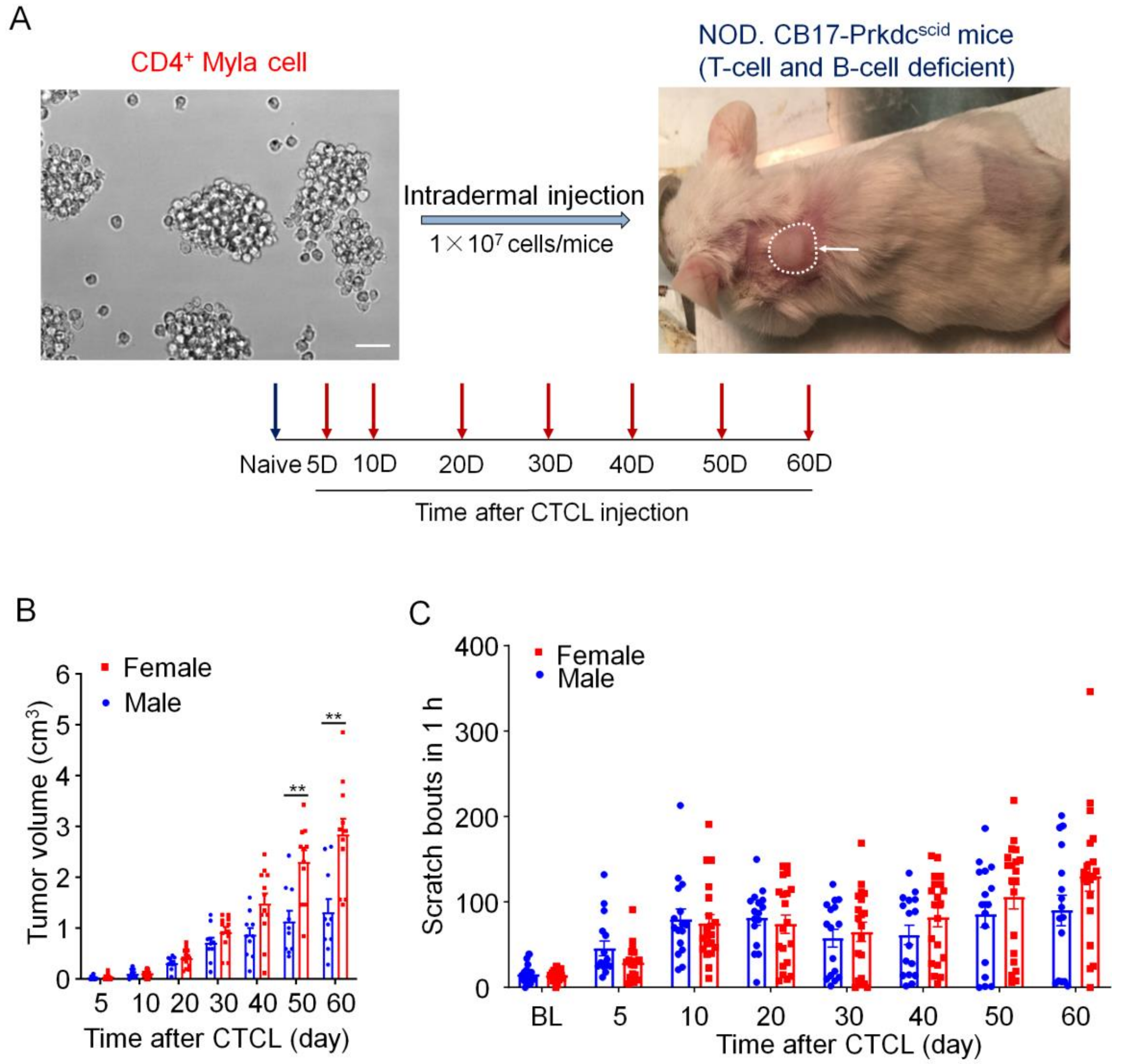

Supplemental Figure 1. Characterization of CTCL-induced tumor growth and itch in males and females. (**A**) Schematic of CD4+ Myla cell culture, intradermal inoculation on the back of the NOD. CB17-Prkdc^{scid} mice, and behavioral assessment of itch. Scale bar: 50 µm. (**B**) Time course of tumor growth in male and female mice. $F_{(6, 118)} = 11.99$, $P<0.0001$. (**C**) Time course of scratch bouts in male and female mice. $F_{(7, 238)} = 1.588$, $P=0.1397$. Data are expressed as mean ± SEM, Two-way ANOVA with Bonferroni post-hoc test. $*P < 0.05$, $**P < 0.01$, n = 10-12 mice per group.

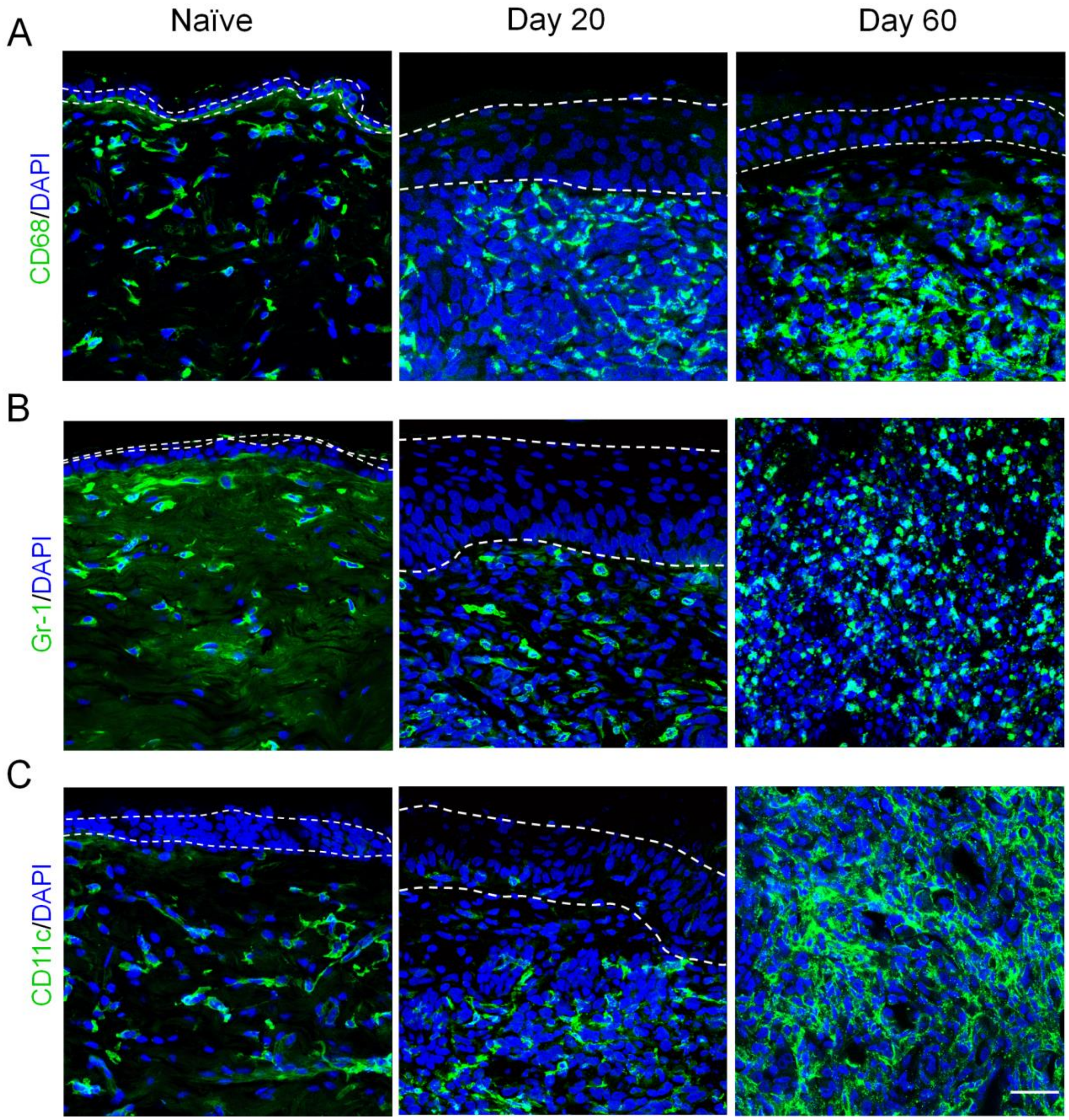

Supplemental Figure 2. CTCL is associated with accumulation of macrophages, neutrophils, and dendritic cells in the tumor-bearing skins on Day 20 and Day 60. Immunostaining was conducted for CD68 (**A**), Gr-1 (**B**), and CD11c (**C**) in naive and CTCL skins (Day 20 and Day 60). The dotted lines show the epidermis; scale bar: 25 μm. See quantification in Figure 1, I-K. Note that the day 20 image in Figure 1E appears again in Supplemental Figure 2B.

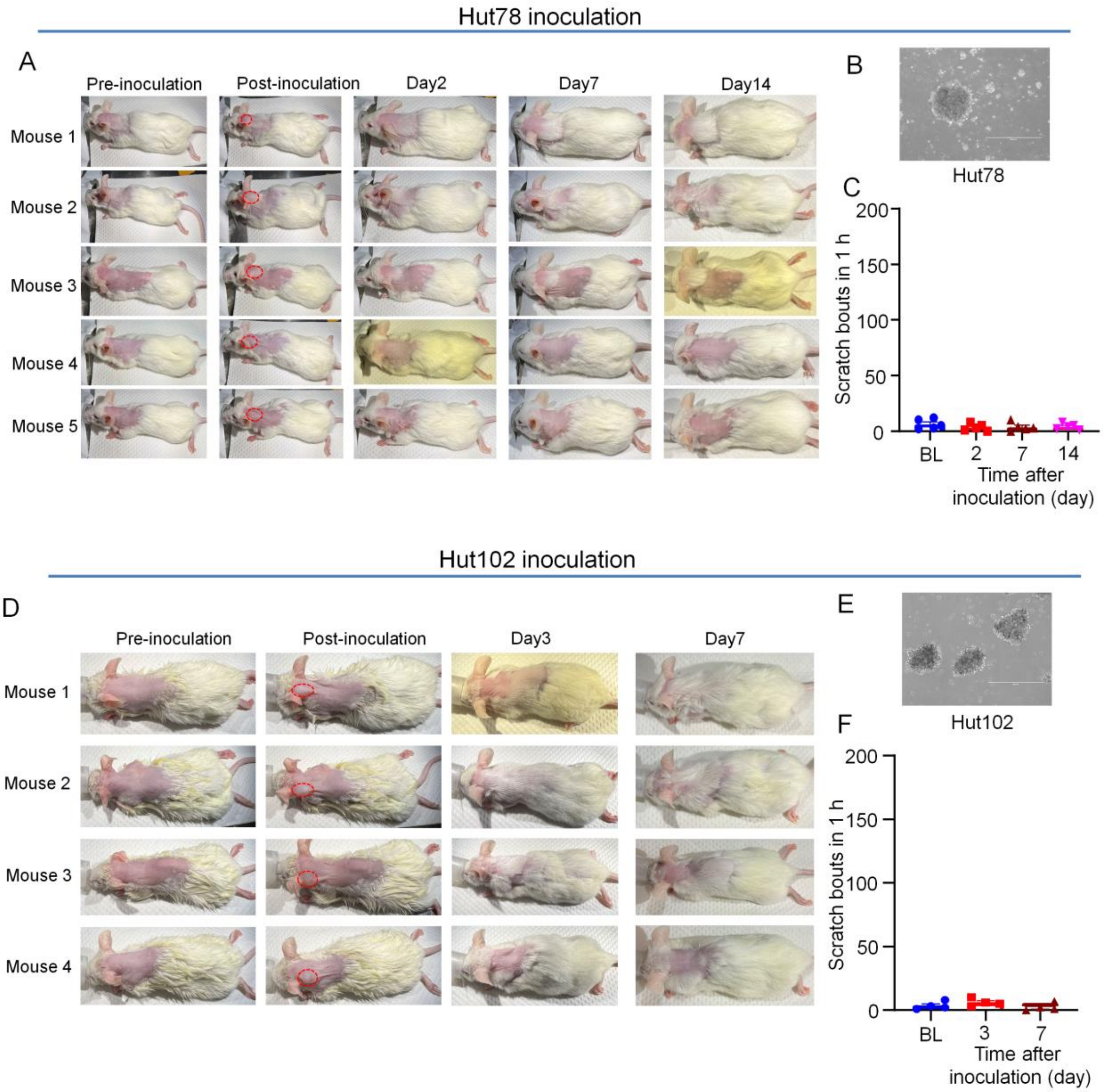

Supplemental Figure 3. Inoculation of Hut78 and Hut102 cells fails to induce tumor growth and itch in skid mice. (**A**) Images of 5 mice with back skins inoculated with Hut78 cells at different times. (**B**) Image of tissue Hut78 cells in culture. Scale, 400 µm. (**C**) Scratch bouts at different times after inoculation. $F_{(3, 16)}$=0.7173, *P*=0.5560. (**D**) Images of 4 mice with back skins inoculated with Hut102 cells. (**E**) Image of tissue Hut102 cells in culture. Scale, 400 µm. (**F**) Scratch bouts at different times after inoculation. $F_{(2, 9)}$=1.393, *P*=0.2972. Hut78 and Hut 102 are two malignant cell lines generated from CTCL patients. Tumor cells were injected into back skin at the centration of 1 x 10^5 cells/µl for a total of volume of 100 µl. Data are expressed as mean ± SEM, Two-way ANOVA with Bonferroni post-hoc test.

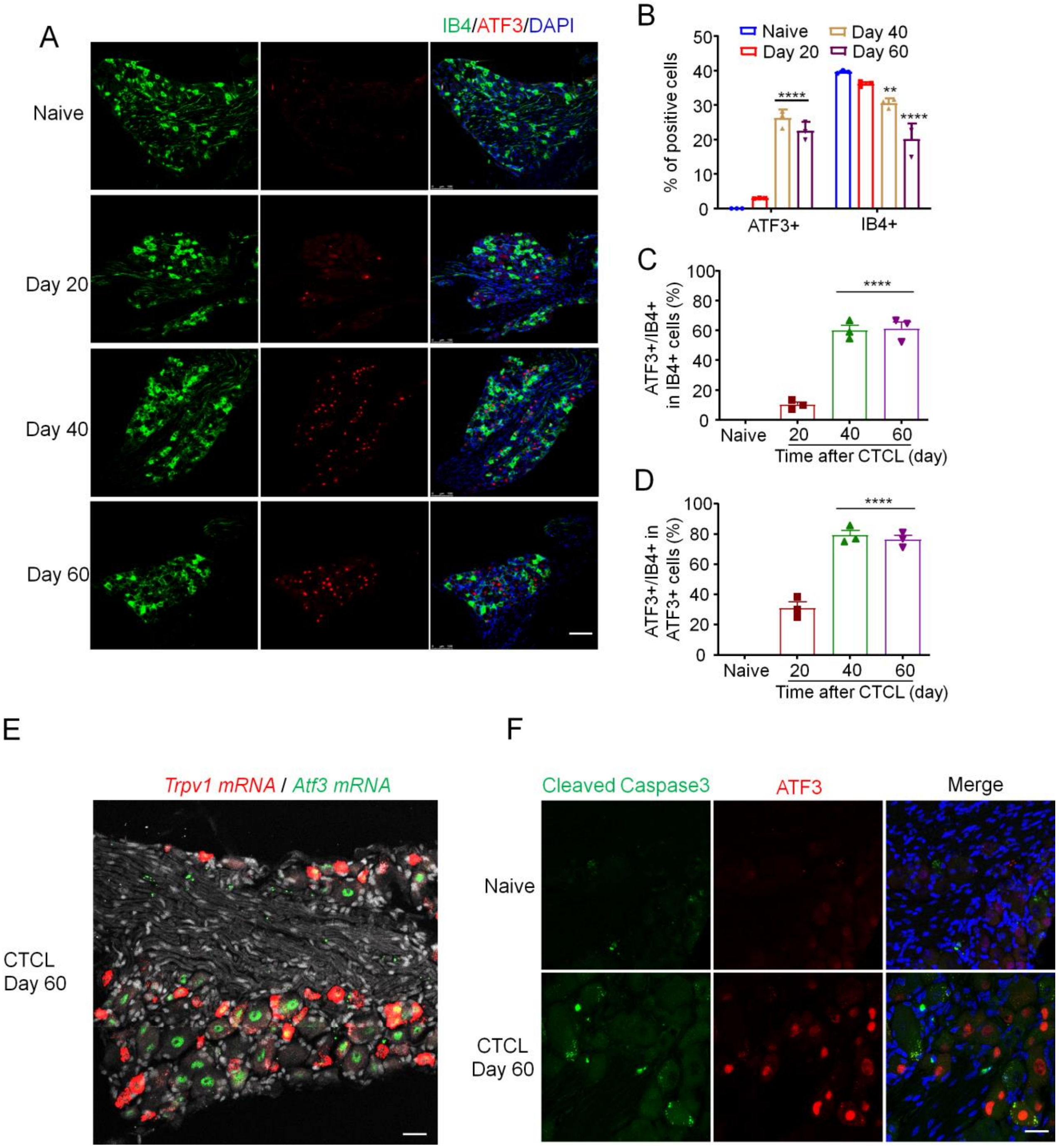

Supplemental Figure 4. Characterization of ATF3, IB4, *Trpv1*, and cleaved caspase 3 expression in DRG of CTCL mice. (**A**) Double immunostaining for ATF3 and IB4 in the cervical DRG from naive mouse and CTCL mouse at day 20, 40, and 60; scale bar: 100 μm. (**B-D**) Percentages of DRG neurons single-labeled with ATF3 and IB4 (B) and double-labeled with ATF3/IB4 in the IB4 population (C) and ATF3 population (D) in the cervical DRG from CTCL mice. Data are expressed as mean ± SEM. One-Way (C,

D) or Two-way ANOVA (B) followed by Bonferroni post-host comparison. n = 3 animals/group. (B) $F_{(3, 12)}$=125.8, *P*<0.0001. (C) $F_{(3, 8)}$=116, *P* < 0.0001. (D) $F_{(3, 8)}$ = 167.9, *P*<0.0001. ***P* < 0.01, *****P* < 0.0001 *vs* naive group. (**E**) In situ hybridization for double staining of *Trpv1* (red, RNAscope) and *Atf3* (green, RNAscope) in DRG of CTCL-Day 60 mice. Scale bar: 50 μm. (**F**) Double immunostaining of ATF3 (red) and cleaved Caspase 3 in DRG of CTCL-Day 60 mice. Scale bar: 50 μm. Note day 60 image in Figure 2A appears again in Supplemental figure 4A.

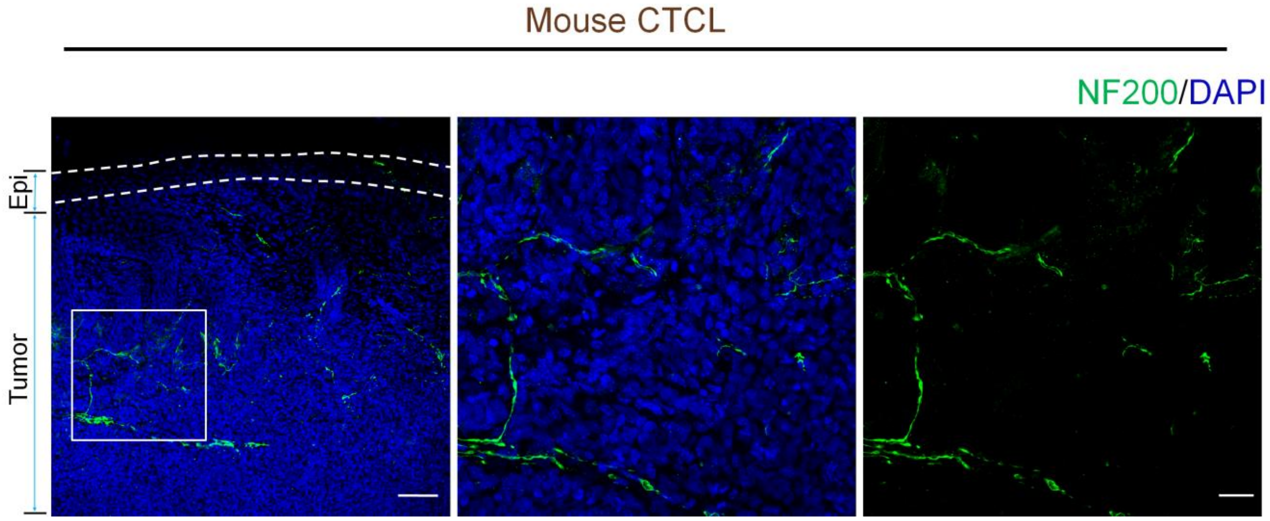

Supplemental Figure 5. Immunostaining for NF200 in the lymphoma from CTCL mouse at Day 20. The dotted lines show the epidermis (Epi). The right two panels are the high magnifications of the box area from the left panel; scale bar: 75 μm (left panel), 25 μm (right panel).

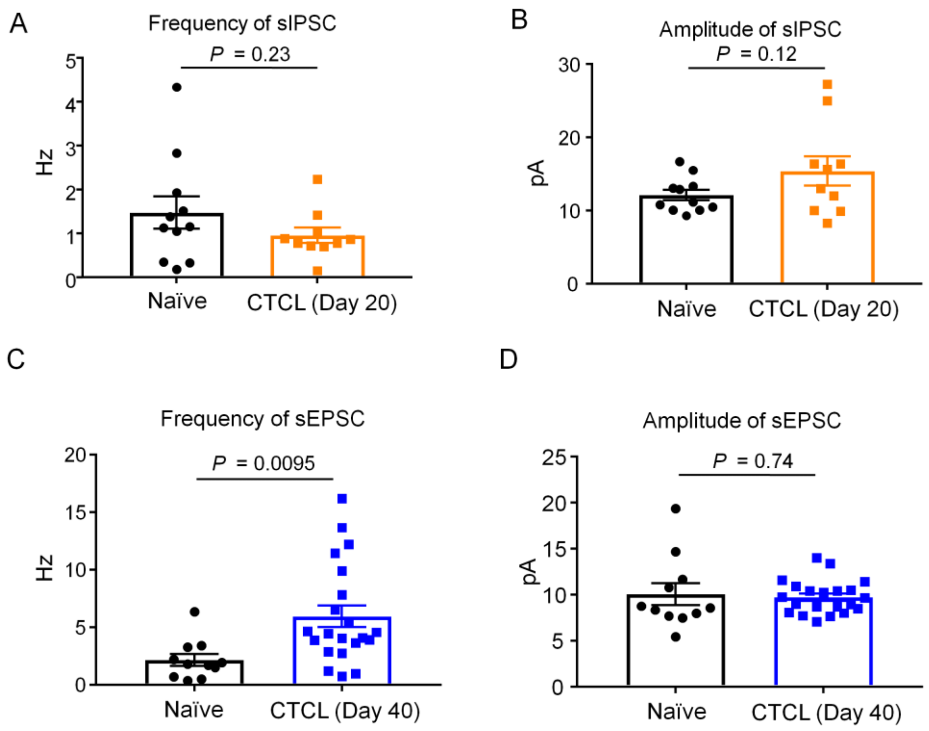

Supplemental Figure 6. Characterization of synaptic transmission in spinal cord slices from naïve and CTCL mice. (**A, B**) Spontaneous IPSC (sIPSC) frequency (A) and amplitude (B) recorded from spinal cord neurons of CB17 naïve and CTCL mice (Day 20). There are no significant differences in the sIPSC frequency and amplitude between control group (n= 11) and CTCL group (n=10). (**C, D**) Spontaneous EPSC (sEPSC) frequency (C) and amplitude (D) recorded from spinal cord neurons of CB17 naïve and late-phase CTCL mice (Day 40). Late-phase CTCL is associated with significant increase in sEPSC frequency but not amplitude. n = 11 neurons (naïve) and n = 21 neurons (CTCL). Data are expressed as Mean ± SEM. Unpaired t-test. Neurons were collected from 3-4 mice from each group.

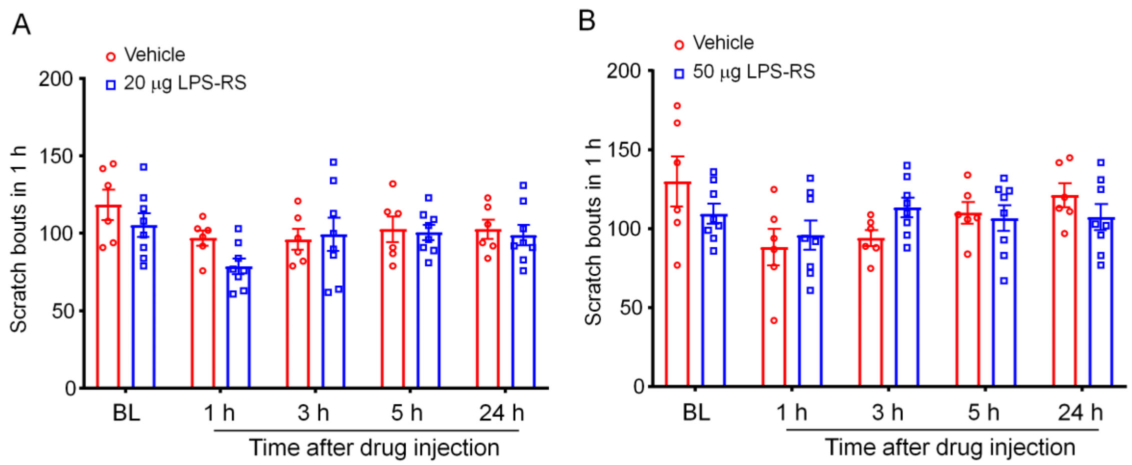

Supplemental Figure 7. Intratumoral injection of TLR4 antagonist fails to reduce CTCL-induced pruritus. (**A**) Effects of 20 µg of LPS-RS on pruritus on CTCL-Day 27. Two-Way ANOVA (P=0.1586, $F_{(1, 60)}$=2.038). (**B**) Effects of 50 µg of LPS-RS on pruritus on CTCL-Day 37. Two-Way ANOVA (P=0.7029, $F_{(1, 60)}$=0.1469); n = 6 mice for vehicle treatment, n = 8 mice for LPS-RS treatment.
